# Supplementary material for: Factors influencing the early initiation of breast feeding in public primary healthcare facilities in Northeast Nigeria: a mixed-method study
Source: BMJ Open. 2020 Apr 20;10(4):e032835. doi: 10.1136/bmjopen-2019-032835 (PMC7204917; doi:10.1136/bmjopen-2019-032835)
Supplement: Author's manuscript [file bmjopen-2019-032835.draft_revisions.pdf]

BMJ Open is committed to open peer review. As part of this commitment we make the peer review history of every article we publish publicly available.

When an article is published we post the peer reviewers' comments and the authors' responses online. We also post the versions of the paper that were used during peer review. These are the versions that the peer review comments apply to.

The versions of the paper that follow are the versions that were submitted during the peer review process. They are not the versions of record or the final published versions. They should not be cited or distributed as the published version of this manuscript.

BMJ Open is an open access journal and the full, final, typeset and author-corrected version of record of the manuscript is available on our site with no access controls, subscription charges or pay-per-view fees (http://bmjopen.bmj.com).

If you have any questions on BMJ Open's open peer review process please email info.bmjopen@bmj.com

BMJ Open

Factors influencing the early initiation of breastfeeding in public primary health care facilities in Northeast Nigeria: A mixed methods study

Journal:	*BMJ Open*
Manuscript ID	bmjopen-2019-032835
Article Type:	Research
Date Submitted by the Author:	17-Jul-2019
Complete List of Authors:	Shobo, Olukolade; Society for Family Health<br>Umar, Nasir; London School of Hygiene and Tropical Medicine Faculty of Infectious and Tropical Diseases, Disease Control<br>Gana, Ahmed; Gombe State Primary Health Care Development Agency<br>Longtoe, Peter; Society for Family Health<br>Idogho, Omokhudu; Society for Family Health<br>Anyanti, Jennifer; Society for Family Health
Keywords:	Health policy < HEALTH SERVICES ADMINISTRATION & MANAGEMENT, Quality in health care < HEALTH SERVICES ADMINISTRATION & MANAGEMENT, PRIMARY CARE, NEONATOLOGY

SCHOLARONE™
Manuscripts

Factors influencing the early initiation of breastfeeding in public primary health care facilities in Northeast Nigeria: A mixed methods study

Corresponding Author:

1. Olukolade George Shobo (Society for Family Health, 8 Port Harcourt Crescent, Area 11, Garki, Abuja. Email: shoboolukolade@gmail.com)

Coauthors:

2. Nasir Umar (London School of Hygiene and Tropical Medicine, London. Email: nasir.umar@lshtm.ac.uk)
3. Gana Ahmed (Gombe State Primary Health Care Development Agency. Gombe State. Email: ahmedgana567@yahoo.com)
4. Longtoe Peter (Society for Family Health, 8 Port Harcourt Crescent, Area 11, Garki, Abuja. Email: plongtoe.com)
5. Omokhudu Idogho (Society for Family Health, 8 Port Harcourt Crescent, Area 11, Garki, Abuja. Email: oidogho@sfhnigeria.org)
6. Jennifer Anyanti (Society for Family Health, 8 Port Harcourt Crescent, Area 11, Garki, Abuja. Email: janyanti@sfhnigeria.org)

ABSTRACT

Introduction

The early initiation of breastfeeding is a high-impact intervention that gives newborns the best chance of survival. We assess the barriers and facilitators influencing the practice of early breastfeeding of newborns in public primary healthcare facilities (PHCs) in Northeast Nigeria, to influence health investment decisions in the region.

Method

We used a mixed methods approach, combining quantitative and qualitative study designs. We conducted case-observation of childbirths and interviewed mothers and birth attendants one hour after childbirth. The analysis for the quantitative arm was done with SPSS version 23. For the qualitative arm, we transcribed the audio files, coded the texts, and categorized them using thematic analysis.

Result

Thirty-nine percent of mothers did not breastfeed their newborns within one hour of birth in our study. Some themes that describe the barriers to early breastfeeding in public PHCs are: birth attendants' unwillingness or inability to accommodate mothers' safe traditional practices, ineffective rooming-in practices, and a lack of privacy and proper visiting-hour policy in public

For peer review only

PHCs. Under one theme, we find that pregnant women denied safe traditional birth practices during delivery are five times more likely not to breastfeed newborns within the first hour of birth (RR=4.5, 95% CI=1.2-17.1) compared to pregnant women allowed these practices.

Conclusion

Close to four of every 10 newborns in public PHCs in Northeast Nigeria do not breastfed in the first hour of birth. This doubles their risk of death in the first 28 days. Stakeholders must increase their focus on the breastfeeding practices in the public PHCs. This will improve the survival of newborns and impact of their investments.

STRENGHTS AND LIMITATIONS OF THE STUDY

- We used a mixed methods study design to provide a richer understanding of the factors that affect newborn breastfeeding practice in public PHCs in resource poor settings.
- We used purposive sampling techniques, focusing on information-rich public PHCs. This improved the chances of observing a high number of childbirths, and identifying typical implementation issues associated with the study aim, during the study period.
- Our study findings are not causal
- The study findings do not generalize to secondary and tertiary health facilities.
- The study involved public PHCs in a resource poor setting and the findings have limited generalizability to PHCs located in places not similar to our study setting.

INTRODUCTION

Every year about 77 million (50%) newborns do not get breastfed in the first hour of birth globally[1]. This leaves them vulnerable to diseases and death[1–3]. Newborn deaths continues to account for close to half of all under-5 mortalities across the world[4]. The early initiation of breastfeeding which means breastfeeding a newborn within one hour of birth[5], is a high-impact intervention[4,6,7] that gives newborns the best chance of survival[8]. It also provides them long-term health benefits[9,10]. The Northeast region of Nigeria has one of the highest newborn mortalities in the world[11–13]. The early initiation of breastfeeding will reduce the risk of these newborn deaths by about a third[14,15].

For peer review only

The practice of early breastfeeding of newborns differs between and within countries[16,17]. For instance, it ranges between 17% to 95% for countries in sub-Saharan Africa [18,19]. In Nigeria, only about 35% of newborns get breastfed within the first hour after birth [20,21]. In the Northeast region of the country, only about 40% of mothers commence the breastfeeding of newborns in the first hour after childbirth[21]. In Gombe State, the estimate is 49%[22]. In the rural areas of the country, mothers are more likely not to practice it at all[20]. The mothers' age, level of education, and socioeconomic status are factors that influence the pattern of early initiation of breastfeeding in the general population. Others are maternal and newborn health problems, childbirth method, family support, availability of supplements, and maternal preference[21,23–25].

To improve early breastfeeding and avert newborn deaths, one approach is for more births to occur in health facilities[14,26–28]. Having a birth in a health facility improves the chance that a newborn will breastfeed early[21]. Evidence however suggests that poor birthing practices occur in health facilities which can disrupt the early start of breastfeeding[17,29–31]. While international recommendations for improving breastfeeding practices in health facilities exist[32], the problems faced by health facilities around the practice context specific[33,34]. Researches focusing on exploring and understanding these specific birthing practices in health facilities are now emerging[18,29].

The Northeast region of Nigeria is witnessing an increase in health investments by governments and non-governmental organizations[35–38]; aimed at reducing maternal and newborn deaths through improved access to quality childbirth and newborn care services in primary health care hospitals (PHCs)[35,36]. In Gombe State in Northeast Nigeria for instance, the government is implementing a Village Health Worker program that improves access to quality obstetric and newborn care services in public PHCs, through community-based demand generation activities. Primary health care facilities are better positioned to deliver high-impact newborn interventions in Nigeria. They make up 88% of health facilities in the country. In the Northeast, there are 5086 public PHCs, and they make up 87% of the health facilities in the region[39,40]. Understanding the factors that influence the quality and uptake of life-saving newborn care services in these public PHCs is important for improving the effectiveness of the health investments in the region.

In this study, we assess the barriers and facilitators influencing early breastfeeding of newborns in public PHCs in Northeast Nigeria. Most studies assessing breastfeeding practice in health facilities have used secondary data from demographic health surveys[21,24,25,41]. Recall bias and lack

For peer review only

of disaggregation between secondary and public health facilities limits the use of their findings[42]. A recent quantitative study in Bangladesh shows that about 43% of mothers in PHCs in the country do not breastfeed their newborns within one hour of birth. It did not assess the contextual implementation issues that influence the practice[31]. The literature around the practice in PHCs in Africa is also lacking. Our approach uses a broader study design that helps to develop a deeper understanding of early breastfeeding practices in public PHCs in Northeast Nigeria[43,44]. We believe our findings will contribute to the discussions about health investments and strategies for improving newborn care in the region, and settings similar to it.

METHOD

Study Design

We use a mixed methods approach for this study. Mixed methods combine quantitative and qualitative study designs to deepen how we understand a research phenomenom[45,46]. In this study, we use the approach to better understand the factors that influence the early initiation of breastfeeding in public PHCs[45]. We conducted the quantitative arm over four weeks in December 2017, and the qualitative arm over one week in November 2018.

Study Setting

The study is set in Gombe State, located in the center of Northeast Nigeria on latitude 9”30’ and 12”30’N, Longitude 8”5’and 11”45’E. It borders Borno, Yobe, Adamawa, Taraba, and Bauchi States. It has 11 Local Government Areas (LGAs) and 114 political wards[47]. There are 603 health facilities across the 11 LGAs in the State, 530 of which are public PHCs[40]. Of the 530 public PHCs in the State, 114 are designated as priority PHCs. The majority (34%) of staff in the PHCs have no medical training[48]. The picture of maternal and newborn health care in the state is comparable to other high mortality settings in sub-Saharan Africa[48].

PHC Selection

Quantitative arm: We selected 10 of the 114 priority PHCs using purposive sampling technique. The 10 had the most deliveries per day on the average, thus suited to provide rich information on our study objective. The budget for the study dictated the number of health facilities chosen.

Qualitative arm: We selected three of the 10 priority PHC in the quantitative arm of the study. The three had the most deliveries per day on the average. The intensity of the childbirths in the

For peer review only

three PHCs and over the study period, will illuminate the barriers and facilitators of early initiation of breastfeeding in public PHCs in the region[49].

Subject Selection

We included all mothers who delivered in the selected PHCs and gave their consent to take part in the study in both arms of the study. For the qualitative arm: we also recruited all consenting healthcare providers who attended deliveries; and the mothers with a live birth for interviews.

Instrument and data collection

Quantitative arm: Trained nurses and nurse-midwives completed the assessment tool. They observed and documented the time of events from when a pregnant woman in labor entered the health facility to when she leaves after childbirth or referral. During data collection, they only observed and did not take part or comment in the care offered to the pregnant woman. We only told them to intervene or offer help during a life-threatening situation to the mother and/or baby.

The health facility staff and pregnant women knew of the nurse and nurse-midwives but were unaware of what was being observed. Data on the cadre of the health worker, events during the first to third stage of labor, and newborn care activities in the first hour after birth were collected. The nurses and nurse-midwives were available for 24hours in each of the health facilities throughout the study period on a shift schedule. They observed all deliveries.

Qualitative arm: The data collection procedure for the qualitative arm is like that of the quantitative arm. The difference is that observation started for each pregnant woman during the second stage of labor and ended one hour after childbirth. The assessment tool used for observation is an extract from the tool used for the quantitative arm. A short interview of the health workers and the mothers occurred one hour after childbirth. We designed the interview questions to be straight to the point with a few follow-up questions around why breastfeeding started or did not. We kept the questions short keeping in mind the workload of the attending health worker and the fatigue of the mother because of labor.

Analysis

Quantitative arm: We analyzed the data using SPSS version 23. We present nominal variables as percentages. We also determined associations and relative risks between initiation of

For peer review only

breastfeeding and predictor categorical variables using two-by-two contingency tables. To assess significant associations, we used Fisher's or Pearson's Chi-Squared test as appropriate

Qualitative arm: We transcribed the interviews from their audio files and analyzed the data with Saturate, an online qualitative software. Two people on the team analyzed a subset of the data and generated codes using a grounded theory approach. The two people then came together to review and agree on the codes generated and their meaning, eliminating less useful codes. We then analyzed the rest of the dataset using the agreed code-framework. We generated themes from the codes using an inductive approach. To analyze the respondent's profile, we used SPSS version 23.

For peer review only

Patient and Public Involvement Statement

Resources for PPI was unavailable, so we could not involve patients. The development and dissemination of a policy brief of the study findings will involve patients.

RESULT

Respondents' Profile

Observation of 393 mothers occurred for the quantitative arm and 27 for the qualitative arm. We also interviewed all 27 mothers and 16 health workers one hour after childbirth under the qualitative arm. The childbirths were through spontaneous vaginal delivery.

Table 1. Respondents' profile

Study participant	Quantitative arm<br>%<br>n=393	Qualitative arms<br>%<br>n=27
Age		
<15	0	3
15 - 24	53	63
25 - 34	36	30
35+	11	4
Gestational age in weeks		
Mean	41 (SD=12)	38(SD=0.6)
Mode	38	38
Median	38	38
Parity	20	33
Nulliparous	80	67

Multiparous		
Attendant health worker during labor and delivery		
nurse/midwife	4	0
junior community health extension worker	36	28
community health extension worker	18	15
environmental health assistant/technician/officer	28	19
hospital assistant	10	19
others (specify)	4	19
Health attendant's sex		
male	1	0
female	99	100
Initiated breastfeeding within the first hour after birth		
no	39	37
yes	61	63

For peer review only

Most (54%) of the women observed under the quantitative arm of the study were between 15 and 24 years old. Their mean gestational age was 41weeks (SD=12). The commonest gestational age was 38 weeks. Only 4% of the women had a nurse or midwife attend to them during the second and third stage of labor. Junior Community Health Extension Workers (JCHEWs) attended the majority (36%) of the deliveries. Environmental health workers and hospital assistants attended close to two-fifths of the deliveries. Thirty-nine percent of the mothers did not breastfeed in the first hour after delivery.

Most (63%) of the women observed under the qualitative arm of the study were between 15 and 24 years old. Their mean gestational age was 38weeks (SD=0.6). Junior Community Health Extension Workers attended the majority (28%) of the deliveries. Environmental health workers and hospital assistants attended close to two-fifths of the deliveries. Thirty-seven percent of the mothers did not breastfeed in the first hour after delivery (Table 1).

Knowledge of time to initiate breastfeeding

The qualitative arm shows that health workers know when mothers should breastfeed newborns. They attributed their knowledge to on-the-job training sessions on newborn care had with visiting clinical-mentors. They believe their knowledge is sustained via peer-to-peer discussions while on the job and during staff meetings. One Junior Community Health Extension Worker (JCHEW) said:

"breast feeding should commence immediately after birth …we have clinical mentors that visit and remind us of these things. We also have staff meetings where we remind ourselves of these practices" (birth attendant 1)

The word "immediately" is more used by junior health workers to describe when breastfeeding should start. The experienced health workers are more specific. breastfeeding should start. They also link the time breastfeeding should start with its benefits. The quote below is a typical response from a Senior CHEW when she was asked when breastfeeding should start.

"It is very important because it helps the child to suck the yellowish nutrient in the breast milk. It boosts the child's immunity. It also helps the mother's uterus to shrink and close …helping to stop bleeding. It should start by 30min to 1hour after delivery" (birth attendant 2).

Barriers to early breastfeeding

Birth attendants' unwillingness or inability to accommodate mothers' safe traditional practices

The quantitative study shows that pregnant women denied safe traditional birth practices during the second and third stages of labor are five times more likely not to breastfeed within the first hour (RR=4.5, 95% CI=1.2-17.1) compared to pregnant women allowed these practices. Not being attended to by a skilled birth attendant (RR=1.2; 95% CI=0.9-1.5), experiencing delays in receiving care (RR=0.7, 95% CI=0.5-1.03), not having birth attendants communicate with the pregnant woman (RR=0.7; 95% CI=0.3-1.8), being attended to by a staff with poor attitude (RR=0.7; 95% CI=0.4-1.1), or not being allowed to give birth in a preferred position (RR=0.9; 95% CI=0.7-1.2) did not influence breastfeeding in the first hour after birth (Table 2).

Table 2: Association between early breastfeeding and predictor variables in the study

Variable	Response	Women who didn't initiate breastfeeding within 1hr after birth		
		%<br>n=154	*Relative risk<br>95% CI*	*p value*
birth attendant had received training on newborn care	No	49	1.2<br>(0.9-1.5)	0.2
	Yes	51		
there were delays in providing care	No	90	0.7<br>(0.5-1.03)	0.2
	Yes	10		
Communication was easy and frequent between woman and birth attendant	No	2	0.7<br>(0.3-1.8)	0.5
	Yes	98		

For peer review only

Poor staff attitude	No	96	0.7 (0.4-1.1)	0.2
	Yes	4		
woman denied some safe traditional childbirth practices*	No	99	4.5 (1.2-17.1)	0.003***
	Yes	1		
birth attendant determined the birth position	No	41	1 (0.8-1.3)	1
	Yes	59		
woman was allowed to give birth in the position she preferred**	No	37	0.9 (0.7-1.2)	0.5
	Yes	63		
the labor room was clean and comfortable	No	18	0.98 (0.7-1.4)	0.9
	Yes	82		

**n=154, **n=150, ***Fishers X^2*

When mother's state of health after childbirth isn't an issue, a typical response given by some mothers for not breastfeeding in the first hour was the need to first wash the breast or have a bath to feel clean.

"he (the baby) has to exercise patience until we get home (before he is breastfed). I can't breastfeed him before I take my bath" (mother 1)

The quantitative study suggests that the mother's need to be clean may not be influenced by the cleanliness of the environment. initiating breastfeeding is not related to the cleanliness and comfort provided by the delivery room. Mothers who had their babies when the labor room was clean and comfortable were just as likely not to initiate breastfeeding early as those who had their babies when the labor room wasn't (RR=0.98; 95% CI 0.7-1.4; p=0.9).

Poor management of mothers' post-delivery state of health

Post-delivery pains and fatigue are barriers to breastfeeding within the first hour after birth. Even when mothers show a good knowledge of when breastfeeding should start, some still express the need to regain strength and wellness first before they breastfeed the newborn.

"breastfeeding should commence immediately after birth… I did not commence it because I was feeling after pains. The health worker said she will bring the baby to suck. I told her to allow me to have some relief" ZA (mother 2)

The quantitative study shows that pregnant women not encouraged to consume fluids or food at least once during labor are twice as likely not to breastfeed within the first hour compared with

For peer review only

those encouraged to do so (RR=2.1; 95% CI=1.5-3; p=0.001). There is no evidence from the study that blood loss greater than 500mls during labor and delivery influence early breastfeeding (p=1).

Manpower shortages

The qualitative study shows that shortage of health workers in PHCs introduces a delay in carrying out newborn care activities. Sometimes, health workers have to attend to other ill patients when there is no one else to assist. When we asked some health workers why a mother under their care did not breastfeed early, a typical response given was:

““You see, if you have someone that will assist you, you will assign the person to carry the baby to the mother and initiate the breastfeeding, or weigh the child, or apply chlorhexidine to the baby's cord or any other thing needed while you continue with the remaining work and management of others …but most of the time you are on duty alone. It is because we have shortage of manpower here in this facility" (birth attendant 3)

The manpower shortage also affects rooming-in in the PHCs. Placing the mother and newborn in the same room after delivery is rooming-in[50]. Sometimes, the health worker has to clean and make this room ready for the mother and newborn. When there is a shortage of staff, this delays the transfer of the mother and newborn to the rooming-in room, and affects early breastfeeding.

"I wanted to transfer the mother and baby to the postnatal ward first before she commences breastfeeding. The room is not set. I have to clean and make the room so she is comfortable to commence breastfeeding" (birth attendant 4)

Ineffective rooming-in practices

When rooming-in happens, the quantitative study reveals that mothers who do not have skin-to skin contact with their newborns in the first hour after birth are twice as likely not to breastfeed early, compared to mothers who did (RR=2.3, 95% CI=1.8-2.8; p<0.001). While the qualitative study shows that delay in rooming-in also delays early breastfeeding, the quantitative study further reveals that just keeping the mother and newborn in the same room (rooming-in) without skin-to-skin contact has no influence on early breastfeeding (RR=2.6; 95% CI=2.3-2.9; p=0.059).

Lack of privacy and proper visiting-hour policy in the PHCs

For peer review only - http://bmjopen.bmj.com/site/about/guidelines.xhtml

The PHCs have open rooming-in rooms that doesn't guarantee privacy. There are also no defined visiting hours in the PHCs. The qualitative arm of the study shows that male and female relatives visit the new mother in the hospital after childbirth. During the visit, the relatives pray for the newborn baby and congratulate the mother. Some relatives sit around after prayers for long. When relatives come visiting, the mothers have to dress up to receive them and do not breastfeed during this time. The birth attendants also delay supporting the mother to breastfeed. One birth attendant suggested that addressing the issue puts them in bad light in the community.

"you know, the people in the community have a unique character or attitude. The moment you try to talk to them about this kind of issue they feel you are molesting them or depriving them of coming close to their relatives. They do not know you are trying to ensure their relative (mother and baby) gets what is beneficial to them" (birth attendant 5).

Facilitators of early breastfeeding

Health education during ANC and post-delivery period

From the qualitative study, we find that Antenatal Clinics (ANC) helps pregnant women to learn about breastfeeding newborns within one hour of birth. The knowledge they gain during these clinics influences their behavior after childbirth. Most mothers who practiced early breastfeeding said what they learned from ANC influenced their decision to do so. The typical response they gave is:

"I used to give my children water (after childbirth). I did not know the importance of breastfeeding early. I used to think breast feeding could start at any time of the day (of birth). I started breastfeeding early because the health workers tell us (of the importance of starting breastfeeding immediately after birth) during ANC" (mother 3)

Encouraging and supporting mothers to start breastfeeding after childbirth

From the qualitative study, we find that when the birth attendants encourage some reluctant mothers to breastfeed their newborns within one hour of childbirth; they do.

"I was told (by the health worker) to give. Normally, I won't" (mother 4)

For peer review only

The encouragement process takes the form of a negotiation between the birth attendant and the mother. We find that the information passed by the birth attendants to the mothers around the benefits of early breastfeeding, facilitate early breastfeeding in the PHCs.

"I commenced breastfeeding early because of what the health worker said …she explained how it is important to the health of my baby. That's why I commenced it" (mother 5)

Sometimes, showing the mothers how to place and breastfeed the newborns also facilitates early breastfeeding after childbirth in these health facilities.

For peer review only

DISCUSSION

In our study setting, we find that close to four out of every 10 newborns do not get breastfed within the first hour of birth. This doubles their risk of dying in the first 28 days of their lives[10,15]. Our estimate is four percentage points lower than what researcher observed in PHCs in Asia[31]. It is also higher than what others have estimated amongst mothers who had spontaneous vaginal deliveries (SVD) in some secondary health facilities; and lower than what others have estimated amongst mothers who had SVD in some tertiary health facilities, even in Nigeria[31,51,52]. The mixed results emphasize the influence of context on the early breastfeeding of newborns in different health facilities. It underscores the need for unique interventions to address the problem.

The northeast region of Nigeria has a shortage of skilled health workers. The Boko-Haram insurgency has made this worse[53–55]. It is also worsened by staff absenteeism in PHCs in the region. Only about 35% of employed staff in PHCs in Gombe are likely to be at work on any given day, for example[22]. Studies suggest that a shortage of manpower and a dominant population of unskilled health care workers affects the quality of newborn care in health facilities[17,56–58]. This is not overall consistent with our study findings. We find that unskilled health workers are dominant in our study setting. Also, we find that shortage of manpower is a barrier to early initiation of breastfeeding in the PHCs. We did not find that the skills of the birth attendants influenced the early breastfeeding of newborns. This may be because clinical mentors have trained the birth attendants in our study on newborn care. Educational interventions around support for the breastfeeding of newborns has been found to improve health workers' knowledge, attitude, and compliance with the practice[59,60].

In our study, we find that the mothers denied safe traditional birth practices during deliveries were five times more likely not to breastfeed the newborns than the mothers not denied. This

supports the findings from other settings that shows that the unwillingness of birth attendants to accommodate safe traditional birth practices affects mothers' adoption of supportive care[57,61,62]. This may be because mothers perceive denial of such traditional practices as mistreatment or abuse[57,63]. The recent abuse of women affects their breastfeeding behavior[64]. Although in our study, we found no evidence to support this. Instead we find that birth attendants' attitude, rapport skills, and negligence during the delivery period doesn't affect early breastfeeding practice amongst mothers. The discrepancy in findings may be because the women in our study setting perceive or tolerate abuse differently than women in other settings[65].

Washing of the breast with water after childbirth is a hygiene related practiced in parts of the world[66]. In our study, mothers express a strong need to wash the breast and/or have a bath before breastfeeding newborns. This makes the "need for a bath after childbirth" a major theme for why mothers delay breastfeeding newborns in our study setting. Some health workers also agree that mothers should wash their breasts first before breastfeeding newborns after childbirth[18]. Their inability to help the mothers to wash their breasts or have a bath before breastfeeding may be because there is no running water in the health facility. Only 38% of priority PHCs in Gombe have running water[22]. The need to wash the breast may be deeper than the need for hygiene, nonetheless. Washing of the breast or having a bath after childbirth before breastfeeding newborns has traditional and/or religious undertones in other parts of the world[58,67].

The other barriers that influence early breastfeeding of newborn in PHCs that our study finds are the poor state of mothers' health, delay in skin to skin contact during rooming-in, and poor visiting hour policy in the PHC. These findings reinforces what other studies have reported[5,21,24,33,41,52,58]. We suggest however that the poor state of mothers' health is because of post-delivery fatigue in our study setting. The post-delivery fatigue may be because the mother is famished. The quantitative arm of our study shows that mothers not encouraged to take fluids or eat during the second and third stages of labor are more likely not to breastfeed early after childbirth. There is no evidence from our study that the fatigue may be due to blood loss. We also find that rooming-in is not enough and that skin to skin contact must be deliberate. Mothers that did not have skin to skin contact with their newborns while in the rooming-in room were more likely not to breastfeed than those who did in our study. Our study reemphasizes that the lack of restrictions on relatives visiting the mothers in the post-delivery period impedes breastfeeding[68]. This could be because the mothers don't feel comfortable breastfeeding in front of male relatives or visitors.

For peer review only - http://bmjopen.bmj.com/site/about/guidelines.xhtml

Helping mothers to breastfeed within an hour of birth is an international recommendation practiced by health care providers in most of the world[32,69]. We find that birth attendants in our study setting practice this and the strategy facilitates the early breastfeeding of newborns in public PHCs. This may be because there is an awareness of the recommendation[70]. Our study also reinforces findings from previous studies on the benefits of health education on the timely breastfeeding of newborns[71,72]. We find that health education received by mothers during ANC and the post-delivery period improves the practice of early initiation of breastfeeding in public PHCs, in our study setting

CONCLUSION

There is a 40% chance that babies born in public PHCs in Northeast Nigeria will not breastfed in the first hour of birth. This means they'll miss important nutrition that saves lives. The stakeholders in the region must increase their focus on the breastfeeding practices in the public PHCs. This will improve the survival of newborns and impact of their investments. Instituting policies that protect mothers' privacy; and finding innovative ways to accommodate and promote safe traditional practices in the intrapartum and postpartum period in PHCs will improve the early breastfeeding of newborns in these PHCs. The cadre of birth attendants does not matter once trained on newborn care, although manpower shortage is a problem. The birth attendants must be trained on effective rooming-in to further improve early breastfeeding of newborns in these public PHCs, however.

LIST OF ABBREVIATIONS

PHC (Primary Health Care hospital/facility), ANC (Antenatal Care) VHW (Village Health Workers)

DECLARATIONS

Ethics approval and consent to participate

The Gombe State Ministry of Health Ethics Committee granted ethical approval for the qualitative arm of the study (reference no: MOH/ADM/658/VOL.II/104). Mothers and birth attendants also gave their consent to take part in the study before the interviews. The IDEAS team at the London School of Hygiene and Tropical Medicine (LSHTM) got ethical approval for the quantitative arm of the study from LSHTM (reference 6088).

Funding

For peer review only

The study is funded as part of the evaluation of SFH's MNCH2 project being implemented in Gombe State. The views and opinions expressed in the paper are those of the authors and do not reflect those of SFH and/or GSPHCDA. The intervention is funded through donor grants received by SFH.

Data Availability

Data for the quantitative arm of the study is in a secured database at the London School of Hygiene and Tropical Medicine. Write to Nasir Umar through nasir.umar@lshtm.ac.uk to place a reasonable request for the anonymized version of it. Also, write to Shobo Olukolade, through shoboolukolade@gmail.com to ask for the anonymized transcripts of the qualitative data.

Competing interest

OGS and PL are consultants working for Society for Family Health (SFH), OI and JA are full-time staff of SFH. GA is the Former Executive Secretary of Gombe State Primary Health Care Development Agency. SFH's program in Northeast Nigeria seeks to improve MNCH outcomes in the general population.

Author Contribution

OGS conceived of the study and developed the original draft of the manuscript. OGS analyzed the quantitative data. OGS and PL analyzed the qualitative data. NU, GA, JA, and OI reviewed, edited, and made significant contributions to the development of the final manuscript. All authors read and approved the final manuscript.

Acknowledgement

For peer review only

We thank all the staff of SFH MNCH2 project in Gombe, particularly Mrs. Magdalene Okolo for their contributions to the study. We also thank the field staff who conducted the observations and interviews.

REFERENCES

1. World Health Organization. *Reaching every newborn national 2020 milestones: country progress, plans and moving forward*. (2017).
2. Edmond, K. M. *et al.* Delayed Breastfeeding Initiation Increases Risk of Neonatal Mortality. *Pediatrics* **117**, e380–e386 (2006).
3. Mullany, L. C. *et al.* Breast-Feeding Patterns, Time to Initiation, and Mortality Risk among Newborns in Southern Nepal. *J Nutr* **138**, 599–603 (2008).
4. World Health Organization. *Every newborn: an action plan to end preventable deaths*. (World Health Organization, 2014).
5. WHO | Early initiation of breastfeeding to promote exclusive breastfeeding. *WHO* Available at: http://www.who.int/elena/titles/early_breastfeeding/en/. (Accessed: 17th June 2019)
6. WHO | Newborn care at birth. *WHO* Available at: http://www.who.int/maternal_child_adolescent/newborns/care_at_birth/en/. (Accessed: 14th June 2019)
7. Debes, A. K., Kohli, A., Walker, N., Edmond, K. & Mullany, L. C. Time to initiation of breastfeeding and neonatal mortality and morbidity: a systematic review. *BMC Public Health* **13**, S19 (2013).
8. UNICEF & WHO. *Capture the moment: early initiation of breastfeeding : the best start for every newborn*. (2018).
9. Friedrich, M. J. Early Initiation of Breastfeeding. *JAMA* **320**, 1097–1097 (2018).

For peer review only

10. al, K. J., et. Timing of breastfeeding initiation and exclusivity of breastfeeding during the first month of life: effects on neonatal mortality and morbidity--a ... - PubMed - NCBI. Available at: https://www.ncbi.nlm.nih.gov/pubmed/24894730. (Accessed: 10th June 2019)

11. Federal Ministry of Health. Nigeria Every Newborn Action Plan: A plan to end preventable newborn deaths in Nigeria. (2016).

12. Anyamele, O., Akanegbu, B., Assad, J.-C. & Ukawuilulu, J. Differentials in Infant and Child Mortality in Nigeria: Evidence from Pooled 2003 and 2008 DHS Data. *Advances in Management and Applied Economics* **7**, 73–96 (2017).

13. Morakinyo, O. M. & Fagbamigbe, A. F. Neonatal, infant and under-five mortalities in Nigeria: An examination of trends and drivers (2003-2013). *PLOS ONE* **12**, e0182990 (2017).

14. Tura, G., Fantahun, M. & Worku, A. The effect of health facility delivery on neonatal mortality: systematic review and meta-analysis. *BMC Pregnancy Childbirth* **13**, 18 (2013).

15. Khan, J., Vesel, L., Bahl, R. & Martines, J. C. Timing of Breastfeeding Initiation and Exclusivity of Breastfeeding During the First Month of Life: Effects on Neonatal Mortality and Morbidity—A Systematic Review and Meta-analysis. *Matern Child Health J* **19**, 468–479 (2015).

16. Takahashi, K. *et al.* Prevalence of early initiation of breastfeeding and determinants of delayed initiation of breastfeeding: secondary analysis of the WHO Global Survey. *Scientific Reports* **7**, 44868 (2017).

17. Haile, Z. T. *et al.* The Moderating Effect of Urban Versus Rural Residence on the Relationship Between Type of Birth Attendant and Early Initiation of Breastfeeding in Ghana. *J Hum Lact* **34**, 810–820 (2018).

For peer review only

18. Bee, M., Shiroor, A. & Hill, Z. Neonatal care practices in sub-Saharan Africa: a systematic review of quantitative and qualitative data. *Journal of Health, Population and Nutrition* **37**, 9 (2018).

19. Sobel, H. L., Silvestre, M. A. A., Iii, J. B. V. M., Oliveros, Y. E. & Nyunt-U, S. Immediate newborn care practices delay thermoregulation and breastfeeding initiation. *Acta Paediatrica* **100**, 1127–1133 (2011).

20. Adewuyi, E. O., Zhao, Y., Khanal, V., Auta, A. & Bulndi, L. B. Rural-urban differences on the rates and factors associated with early initiation of breastfeeding in Nigeria: further analysis of the Nigeria demographic and health survey, 2013. *International Breastfeeding Journal* **12**, 51 (2017).

21. Berde, A. S. & Yalcin, S. S. Determinants of early initiation of breastfeeding in Nigeria: a population-based study using the 2013 demograhic and health survey data. *BMC Pregnancy and Childbirth* **16**, 32 (2016).

22. Marchant, T. *Change in maternal and newborn health care interactions between families and frontline workers. Report from Gombe State, Nigeria, 2012-2015*. (London School of Hygiene & Tropical Medicine, 2016).

23. Thulier, D. & Mercer, J. Variables Associated With Breastfeeding Duration. *Journal of Obstetric, Gynecologic & Neonatal Nursing* **38**, 259–268 (2009).

24. Ahmed, A. E. & Salih, O. A. Determinants of the early initiation of breastfeeding in the Kingdom of Saudi Arabia. *International Breastfeeding Journal* **14**, 13 (2019).

25. Liben, M. L. & Yesuf, E. M. Determinants of early initiation of breastfeeding in Amibara district, Northeastern Ethiopia: a community based cross-sectional study. *International Breastfeeding Journal* **11**, 7 (2016).

For peer review only

26. Delivery care. *UNICEF DATA* Available at: https://data.unicef.org/topic/maternal-health/delivery-care/. (Accessed: 11th June 2019)

27. Bohren, M. A. *et al.* Facilitators and barriers to facility-based delivery in low- and middle-income countries: a qualitative evidence synthesis. *Reproductive Health* **11**, 71 (2014).

28. Campbell, O. M. & Graham, W. J. Strategies for reducing maternal mortality: getting on with what works. *The Lancet* **368**, 1284–1299 (2006).

29. WHO | Early initiation of breastfeeding. *WHO* Available at: http://www.who.int/elena/titles/commentary/early_breastfeeding/en/. (Accessed: 10th June 2019)

30. Forster, D. A. & McLachlan, H. L. Breastfeeding Initiation and Birth Setting Practices: A Review of the Literature. *Journal of Midwifery & Women's Health* **52**, 273–280 (2007).

31. Karim, F. *et al.* Initiation of breastfeeding within one hour of birth and its determinants among normal vaginal deliveries at primary and secondary health facilities in Bangladesh: A case-observation study. *PLOS ONE* **13**, e0202508 (2018).

32. WHO | Protecting, promoting and supporting breastfeeding in facilities providing maternity and newborn services. *WHO* Available at: http://www.who.int/elena/titles/full_recommendations/breastfeeding-support/en/. (Accessed: 11th June 2019)

33. Sharma, I. K. & Byrne, A. Early initiation of breastfeeding: a systematic literature review of factors and barriers in South Asia. *International Breastfeeding Journal* **11**, 17 (2016).

34. DiGirolamo, A. M., Grummer-Strawn, L. M. & Fein, S. Maternity Care Practices: Implications for Breastfeeding. *Birth* **28**, 94–100 (2001).

35. Fedreal Ministry of Health. Nigeria Investment case for reproductive, maternal, newborn, child, adolescent health and nutrition: 2017 - 2030. (2017).

For peer review only

36. The Maternal and Neonatal Health Project in North East Nigeria 2012 – 2016 | Society for Family Health, Nigeria. Available at: http://www.sfhnigeria.org/the-maternal-and-neonatal-health-project-in-north-east-nigeria-2012-2016/. (Accessed: 15th June 2019)

37. State Accountability and Quality Improvement Project. *Pact* Available at: https://www.pactworld.org/country/nigeria/project. (Accessed: 16th June 2019)

38. Strengthening the healthcare system in north-east Nigeria - a priority for WHO | WHO | Regional Office for Africa. Available at: https://www.afro.who.int/news/strengthening-healthcare-system-north-east-nigeria-priority-who. (Accessed: 16th June 2019)

39. Makinde, O. A., Sule, A., Ayankogbe, O. & Boone, D. Distribution of health facilities in Nigeria: Implications and options for Universal Health Coverage. *The International Journal of Health Planning and Management* (2018). doi:10.1002/hpm.2603

40. Nigeria Health Facility Registry. Available at: https://hfr.health.gov.ng/facilities/search?_token=r2vSeMamYxcYqkwMtdRF67CgGTWkRNkpYS6QoUQV&facility_type_id=1&state_id=115&lga_id=&ward_id=0&facility_level_id=2&ownership_id=1&operational_status_id=0&registration_status_id=0&license_status_id=0&geo_codes=0&service_type=0&service_category_id=0&facility_name=. (Accessed: 14th March 2019)

41. Bbaale, E. Determinants of Early Initiation, Exclusiveness, and Duration of Breastfeeding in Uganda. *J Health Popul Nutr* **32**, 249–260 (2014).

42. Boerma, J. T. & Sommerfelt, A. E. Demographic and health surveys (DHS): contributions and limitations. *World Health Stat Q* **46**, 222–226 (1993).

43. Kawulich, B. B. Participant Observation as a Data Collection Method. *Forum Qualitative Sozialforschung / Forum: Qualitative Social Research* **6**, (2005).

44. Altmann, J. Observational Study of Behavior: Sampling Methods. *Behaviour* **49**, 227–267 (1974).

For peer review only

45. Schoonenboom, J. & Johnson, R. B. How to Construct a Mixed Methods Research Design. *Kolner Z Soz Sozpsychol* **69**, 107–131 (2017).

46. Denzin, N. K. & Lincoln, Y. S. *The SAGE Handbook of Qualitative Research*. (SAGE, 2011).

47. Gombe State Government. Gombe State Nigeria: History. *Gombe State*

48. Marchant, D. T. Maternal and newborn health care. Baseline findings from Gombe State, Nigeria. (2013). doi:10.17037/PUBS.01126638

49. Patton, M. Designing Qualitative Studies. in *Qualitative evaluation and research methods* 169–186 (SAGE, 1990).

50. Jaafar, S. H., Ho, J. J. & Lee, K. S. Rooming-in for new mother and infant versus separate care for increasing the duration of breastfeeding. *Cochrane Database Syst Rev* CD006641–CD006641 (2016). doi:10.1002/14651858.CD006641.pub3

51. Örün, E. *et al.* Factors associated with breastfeeding initiation time in a Baby-Friendly Hospital. *The Turkish Journal of Pediatrics* **52**, 10–16 (2010).

52. Awi, D. D. & Alikor, E. a. D. Barriers to timely initiation of breastfeeding among mothers of healthy full-term babies who deliver at the University of Port Harcourt Teaching Hospital. *Nigerian Journal of Clinical Practice* **9**, 57-64–64 (2006).

53. Federal Ministry of Health. National Human Resource for Health Strategic Plan: 2008 - 2012. (2008).

54. Premium Times Nigeria. Gombe govt. waives retirement for 100 nurses, midwives due to manpower shortage. (2017). Available at: https://www.premiumtimesng.com/news/more-news/230165-gombe-govt-waives-retirement-100-nurses-midwives-due-manpower-shortage.html. (Accessed: 17th June 2019)

55. Omoluabi, E. *Needs Assessment of the Nigerian Health Sector*. (International Organization for Migration, 2014).

For peer review only

56. Aluko, J. O., Anthea, R. & Marie Modeste, R. R. Manpower capacity and reasons for staff shortage in primary health care maternity centres in Nigeria: a mixed-methods study. *BMC Health Services Research* **19**, 10 (2019).

57. Bohren, M. A. *et al.* The Mistreatment of Women during Childbirth in Health Facilities Globally: A Mixed-Methods Systematic Review. *PLoS Med* **12**, e1001847 (2015).

58. Majra, J. P. & Silan, V. K. Barriers to Early Initiation and Continuation of Breastfeeding in a Tertiary care Institute of Haryana: A Qualitative Study in Nursing Care Providers. *J Clin Diagn Res* **10**, LC16–LC20 (2016).

59. Balogun, O. O. *et al.* Health Facility Staff Training for Improving Breastfeeding Outcome: A Systematic Review for Step 2 of the Baby-Friendly Hospital Initiative. *Breastfeeding Medicine* **12**, 537–546 (2017).

60. Okolo, S. N. & Ogbonna, C. Knowledge, attitude and practice of health workers in Keffi local government hospitals regarding Baby-Friendly Hospital Initiative (BFHI) practices. *Eur J Clin Nutr* **56**, 438–441 (2002).

61. Mannava, P., Durrant, K., Fisher, J., Chersich, M. & Luchters, S. Attitudes and behaviours of maternal health care providers in interactions with clients: a systematic review. *Globalization and Health* **11**, 36 (2015).

62. Chatters, L. M. Religion and Health: Public Health Research and Practice. *Annual Review of Public Health* **21**, 335–367 (2000).

63. Beck, C. T. A Secondary Analysis of Mistreatment of Women During Childbirth in Health Care Facilities. *Journal of Obstetric, Gynecologic & Neonatal Nursing* **47**, 94–104 (2018).

64. Sørbø, M. F., Lukasse, M., Brantsæter, A.-L. & Grimstad, H. Past and recent abuse is associated with early cessation of breast feeding: results from a large prospective cohort in Norway. *BMJ Open* **5**, e009240 (2015).

For peer review only

65. Maya, E. T. *et al.* Women's perspectives of mistreatment during childbirth at health facilities in Ghana: findings from a qualitative study. *Reproductive Health Matters* **26**, 70–87 (2018).

66. Khan, M. E. Shaping Demand and Practices to Improve Family Health Outcomes: Findings from a Quantitative Survey, Volume 3: Bihar. 221

67. Tawiah-Agyemang, C., Kirkwood, B. R., Edmond, K., Bazzano, A. & Hill, Z. Early initiation of breast-feeding in Ghana: barriers and facilitators. *Journal of Perinatology* **28**, S46–S52 (2008).

68. Saxena, M., Srivastava, A., Dwivedi, P. & Bhattacharyya, S. Is quality of care during childbirth consistent from admission to discharge? A qualitative study of delivery care in Uttar Pradesh, India. *PLOS ONE* **13**, e0204607 (2018).

69. UNICEF & World Health Organization. Country Experiences with the Baby-Friendly Hospital Initiatiive. Compendium of Case Studies from Around the World. (2017).

70. Kakrani, V. A., Rathod (Waghela), H. K., Mammulwar, M. S. & Bhawalkar, J. S. Awareness about "Ten Steps for Successful Breastfeeding" among Medical and Nursing Students. *Int J Prev Med* **6**, (2015).

71. Guise, J.-M. *et al.* The Effectiveness of Primary Care-Based Interventions to Promote Breastfeeding: Systematic Evidence Review and Meta-Analysis for the US Preventive Services Task Force. *Ann Fam Med* **1**, 70–78 (2003).

72. de Oliveira, M. I. C., Camacho, L. A. B. & Tedstone, A. E. Extending Breastfeeding Duration Through Primary Care: A Systematic Review of Prenatal and Postnatal Interventions. *J Hum Lact* **17**, 326–343 (2001).

For peer review only

BMJ Open

Factors influencing the early initiation of breastfeeding in public primary health care facilities in Northeast Nigeria: A mixed methods study

Journal:	*BMJ Open*
Manuscript ID	bmjopen-2019-032835.R1
Article Type:	Research
Date Submitted by the Author:	26-Nov-2019
Complete List of Authors:	Shobo, Olukolade; Society for Family Health , Monitoring and Evaluation Department<br>Umar, Nasir; London School of Hygiene and Tropical Medicine Faculty of Infectious and Tropical Diseases, Disease Control<br>Gana, Ahmed; Gombe State Primary Health Care Development Agency, Office of the Executive Secretary<br>Longtoe, Peter; Society for Family Health , Monitoring and Evaluation Department<br>Idogho, Omokhudu; Society for Family Health , Office of the Managing Director<br>Anyanti, Jennifer; Society for Family Health , Office of the Deputy Managing Director
**Primary Subject Heading**:	Health services research
Secondary Subject Heading:	Nutrition and metabolism, Obstetrics and gynaecology
Keywords:	Health policy < HEALTH SERVICES ADMINISTRATION & MANAGEMENT, Quality in health care < HEALTH SERVICES ADMINISTRATION & MANAGEMENT, PRIMARY CARE, NEONATOLOGY

SCHOLARONE™ Manuscripts

I, the Submitting Author has the right to grant and does grant on behalf of all authors of the Work (as defined in the below author licence), an exclusive licence and/or a non-exclusive licence for contributions from authors who are: i) UK Crown employees; ii) where BMJ has agreed a CC-BY licence shall apply, and/or iii) in accordance with the terms applicable for US Federal Government officers or employees acting as part of their official duties; on a worldwide, perpetual, irrevocable, royalty-free basis to BMJ Publishing Group Ltd ("BMJ") its licensees and where the relevant Journal is co-owned by BMJ to the co-owners of the Journal, to publish the Work in this journal and any other BMJ products and to exploit all rights, as set out in our licence.

The Submitting Author accepts and understands that any supply made under these terms is made by BMJ to the Submitting Author unless you are acting as an employee on behalf of your employer or a postgraduate student of an affiliated institution which is paying any applicable article publishing charge ("APC") for Open Access articles. Where the Submitting Author wishes to make the Work available on an Open Access basis (and intends to pay the relevant APC), the terms of reuse of such Open Access shall be governed by a Creative Commons licence – details of these licences and which Creative Commons licence will apply to this Work are set out in our licence referred to above.

Other than as permitted in any relevant BMJ Author's Self Archiving Policies, I confirm this Work has not been accepted for publication elsewhere, is not being considered for publication elsewhere and does not duplicate material already published. I confirm all authors consent to publication of this Work and authorise the granting of this licence.

For peer review only

Factors influencing the early initiation of breastfeeding in public primary health care facilities in Northeast Nigeria: A mixed methods study

Corresponding Author:

1. Olukolade George Shobo (Society for Family Health, 8 Port Harcourt Crescent, Area 11, Garki, Abuja. Email: shoboolukolade@gmail.com)

Coauthors:

2. Nasir Umar (London School of Hygiene and Tropical Medicine, London. Email: nasir.umar@lshtm.ac.uk)
3. Gana Ahmed (Gombe State Primary Health Care Development Agency. Gombe State. Email: ahmedgana567@yahoo.com)
4. Peter Longtoe (Society for Family Health, 8 Port Harcourt Crescent, Area 11, Garki, Abuja. Email: plongtoe.com)
5. Omokhudu Idogho (Society for Family Health, 8 Port Harcourt Crescent, Area 11, Garki, Abuja. Email: oidogho@sfhnigeria.org)
6. Jennifer Anyanti (Society for Family Health, 8 Port Harcourt Crescent, Area 11, Garki, Abuja. Email: janyanti@sfhnigeria.org)

For peer review only

ABSTRACT

Introduction

The early initiation of breastfeeding is a high-impact intervention that gives newborns a better chance of survival. We assess the barriers and facilitators influencing the practice of early breastfeeding of newborns in public primary healthcare facilities (PHCs) in Northeast Nigeria, to influence the planning of programs targeted at improving newborn care in the region.

Method

We used an explanatory mixed methods approach. We conducted case-observation of childbirths and newborn care for the quantitative arm, and interviewed mothers and birth attendants one hour after childbirth for the qualitative arm. The analysis for the quantitative arm was done with SPSS version 23. For the qualitative arm, we transcribed the audio files, coded the texts, and categorized them using thematic analysis.

Result

We observed 393 and 27 mothers for the quantitative and qualitative arms of the study respectively. The quantitative arm shows that 39% of mothers did not breastfeed their newborns within one hour of birth. The qualitative arm shows that 37% of mothers did not breastfeed within one hour of birth. Themes that describe the barriers to early breastfeeding in public PHCs are:

birth attendants' unwillingness or inability to accommodate mothers' safe traditional practices, ineffective rooming-in practices, staff shortages, lack of privacy in the lying-in ward, and poor implementation of visiting-hour policy in public PHCs. The pregnant women denied safe traditional birth practices like chanting, praying, or reading religious books during delivery are five times more likely not to breastfeed newborns within the first hour of birth (RR=4.5, 95% CI=1.2-17.1) compared to pregnant women allowed these practices.

Conclusion

Stakeholders must increase their focus on improving the breastfeeding practices in the public PHCs. Instituting policies that protect mothers' privacy; and finding innovative ways to accommodate and promote safe traditional practices in the intrapartum and postpartum period in PHCs will improve the early breastfeeding of newborns in these PHCs.

STRENGHTS AND LIMITATIONS OF THE STUDY

- We used a mixed methods study design to provide a richer understanding of the factors that affect newborn breastfeeding practice in public PHCs in resource poor settings.
- We used purposive sampling techniques, focusing on information-rich public PHCs. This improved the chances of observing a high number of childbirths, and identifying typical implementation issues associated with the study aim, during the study period.
- Our study findings are not causal.
- The study findings may not generalize to secondary and tertiary health facilities, nor to PHCs located in places not similar to our study setting.

INTRODUCTION

Every year, about 77 million (50%) newborns do not get breastfed in the first hour of birth globally[1]. This leaves them vulnerable to diseases and death[1–3]. Newborn deaths continue to account for close to half of all under-5 mortalities across the world[4]. The early initiation of breastfeeding which means breastfeeding a newborn within one hour of birth[5], is a high-impact intervention[4,6,7] that gives newborns a better chance of survival[8]. It also provides them long-term health benefits[9,10]. The Northeast region of Nigeria has one of the highest newborn mortalities in

For peer review only

the world[11–13]. The early initiation of breastfeeding will reduce the risk of these newborn deaths by about a third[14,15].

The practice of early breastfeeding of newborns differs between and within countries[16,17]. For instance, it ranges between 17% to 95% for countries in sub-Saharan Africa [18,19]. In Nigeria, only about 35% of newborns get breastfed within the first hour after birth [20,21]. In the Northeast region of the country, only about 40% of mothers commence the breastfeeding of newborns in the first hour after childbirth[21]. In Gombe State, the estimate is 49%[22]. In the rural areas of the country, mothers are more likely not to practice it at all[20]. The mothers' age, level of education, and socioeconomic status are factors that influence the pattern of early initiation of breastfeeding in the general population. Others are maternal and newborn health problems, childbirth method, family support, availability of supplements, and maternal preference[21,23–25].

To improve early breastfeeding and avert newborn deaths, one approach is for more births to occur in health facilities[14,26–28]. Having a birth in a health facility improves the chance that a newborn will breastfeed early[21]. Evidence however suggests that poor birthing practices occur in health facilities which can disrupt the early start of breastfeeding[17,29–31]. While international recommendations for improving breastfeeding practices in health facilities exist[32], the problems faced by health facilities around the practice are context specific[33,34], requiring unique responses. Researches focusing on exploring and understanding these specific birthing practices in health facilities are now emerging[18,29].

The Northeast region of Nigeria is witnessing an increase in health investments by governments and non-governmental organizations[35–38]; aimed at reducing maternal and newborn deaths through improved access to quality childbirth and newborn care services in primary health care hospitals (PHCs)[35,36]. In Gombe State in Northeast Nigeria for instance, the government is implementing a Village Health Worker program that improves access to quality obstetric and newborn care services in public PHCs, through community-based demand generation activities. Primary health care facilities are better positioned to deliver high-impact newborn interventions in Nigeria. They make up 88% of health facilities in the country. In the Northeast, there are 5,086 public PHCs, and they make up 87% of the health facilities in the region[39,40]. Understanding the factors that influence the quality and uptake of life-saving newborn care services in these public PHCs is important for improving the effectiveness of the health investments in the region.

For peer review only

In this study, we assess the barriers and facilitators influencing early breastfeeding of newborns in public PHCs in Northeast Nigeria. Most studies assessing breastfeeding practice in health facilities have used secondary data from demographic health surveys[21,24,25,41]. While maternal recall is valid and reliable in establishing the period of initiation of breastfeeding[42], the DHS findings do not provide context concerning supply side factors that influenced the maternal behavior. A recent quantitative study in Bangladesh shows that about 43% of mothers in PHCs in the country do not breastfeed their newborns within one hour of birth. It did not assess the contextual implementation issues that influence the practice[31]. The literature around the contextual issues affecting the practice in PHCs in Africa is also lacking. Our approach uses a broader study design that helps to develop a deeper understanding of early breastfeeding practices in public PHCs in Northeast Nigeria[43,44]. We believe our findings will contribute to the discussions about health investments and strategies for improving newborn care in the region, and settings similar to it.

METHOD

Study Design

We used an explanatory mixed methods approach for this study. Mixed methods study designs deepen how we understand a research phenomenom[45,46]. In the explanatory mixed method type, a first phase quantitative data collection and analysis is followed by the collection of qualitative data, to explain the quantitative result[45]. The mixed-method approach helps us better understand the factors that influence the early initiation of breastfeeding in public PHCs[45]. We conducted the quantitative arm over four weeks in December 2017, and the qualitative arm over one week in November 2018. Budget constraints delayed implementing the qualitative arm. We assume that the time difference between the study arms is not sufficient to change the practice around breastfeeding newborns in the study setting.

Study Setting

We conducted the study in Gombe State, in the center of Northeast Nigeria on latitude 9” 30’ and 12” 30’N, Longitude 8” 5’and 11” 45’E. It borders Borno, Yobe, Adamawa, Taraba, and Bauchi State. It has 11 Local Government Areas (LGAs) and 114 political wards[47]. There are 603 health facilities across the 11 LGAs in the State, 530 of which are public PHCs[40]. Of the 530 public PHCs in the State, the government has designated 114 as priority PHCs. These 114 are Ward Health

For peer review only - http://bmjopen.bmj.com/site/about/guidelines.xhtml

Centers and provide basic emergency obstetric and newborn care services. Nurses, community health workers, community health extension workers (CHEWs), junior CHEWs, and environmental health officers are the main staff of PHCs in Nigeria[48]. Community Health Officer, a Public Health Nurse, three CHEWs, four Nurse/Midwives and one medical Assistant are the main staff of a Ward Health Center[49].

Fifty percent of these priority PHCs have staff trained in providing basic emergency obstetric and newborn care services. About 36% of them have labor rooms and lying-in wards. None has a medical doctor, 4% have at least one nurse, and 19% have at least one midwife. Each PHC has an average of six health workers not categorized as skilled in attending labor and delivery. These include Community Health Extension Workers (CHEWs), environmental health technicians, hospital assistants (cleaners), and students. The majority (34%) of the staff in these PHCs have no medical training[50]. Fewer than half of pregnant women in the State access pregnancy care at least four times as recommended, and only about a third access facility based intra-partum care and/or skilled attendance at birth[50]. Cesarean sections are not conducted in PHCs in the study setting.

PHC Selection

Quantitative arm: We selected 10 of the 114 priority PHCs using purposive sampling technique. The 10 had the most deliveries per day on the average in the six-month period prior to starting the study, thus suited to provide rich information on our study objective. They also had labor wards and lying-in wards. The budget for the study dictated the number of health facilities chosen.

Qualitative arm: We selected three of the 10 priority PHC in the quantitative arm of the study. The three had the most deliveries per day on the average. They also had labor wards and lying-in wards. The intensity of the childbirths in the three PHCs and over the study period, will illuminate the barriers and facilitators of early initiation of breastfeeding in public PHCs in the region[51].

Subject Selection

We included all mothers who delivered in the selected PHCs and gave their consent to take part in the study in both arms of the study. For the qualitative arm: we also recruited all consenting healthcare providers who attended deliveries; and the mothers with a live birth for interviews.

Instrument and data collection

For peer review only

Quantitative arm: We recruited and trained nurses and nurse-midwives who are not a part of the health facility staff to complete the assessment tool. They observed and documented the time of events from when a pregnant woman in labor entered the health facility to when she leaves after childbirth or referral. During data collection, they only observed and did not take part or comment in the care offered to the pregnant woman. We only told them to intervene or offer help during a life-threatening situation to the mother and/or baby. The data collectors used separate assessment tools in cases of twin deliveries.

The health facility staff and pregnant women knew of the nurse and nurse-midwives but were unaware of what aspect of care was being observed. Data on the cadre of the health worker, events during the first to third stage of labor, and newborn care activities in the first hour after birth were collected. The nurses and nurse-midwives were available for 24hours in each of the health facilities throughout the study period on a shift schedule. They observed all deliveries.

Qualitative arm: We also recruited and trained female nurses and midwives who are not part of the health facility staff to observe and complete the assessment tool under this arm of the study. Observation for each pregnant woman started during the second stage of labor and ended one hour after childbirth under this arm of the study. The data collector then interviewed the mother and attendant health worker, after the observation period. The assessment tool used for the observation is an extract from the tool used for the quantitative arm. For the mothers and birth attendants we asked questions around how they felt about the childbirth process and knowledge of when newborn babies should breastfeed during the interview. We also asked the mothers why they chose to or not to breastfeed their newborns within the hour depending on if they did or not. Also, we asked the attending healthcare workers why they think breastfeeding occurred or did not occur within the first hour for each newborn. We also asked the attending healthcare workers about the strategies they used to encourage mothers to breastfeed their newborns within the hour. The data collectors attended every birth in the health facilities and interviewed respondents who could not speak or understand English in Hausa. They interviewed the mother and attending healthcare worker separately. The mothers' interviews occurred at their bedside in the lying-in ward. The data collectors excused relatives or visitors in the lying-in room during interviews.

Analysis

For peer review only

Quantitative arm: We analyzed the data using SPSS version 23. We present nominal variables as percentages. We also determined associations and relative risks between initiation of breastfeeding and predictor categorical variables using two-by-two contingency tables. To assess significant associations, we used Fisher's or Pearson's Chi-Squared test as appropriate

Qualitative arm: We transcribed the interviews from their audio files and analyzed the data with Saturate, an online qualitative software. Two people on the team analyzed a subset of the data and generated codes. The two people then came together to review and agree on the codes generated and their meaning, eliminating less useful codes. The two reviewers held regular face-to-face meetings to discuss their codes. We then analyzed the rest of the dataset using the agreed code-framework developed by the two. We generated themes from the codes using an inductive approach.

Patient and Public Involvement Statement

Resources for patient and public involvement statement was unavailable, so we could not involve patients. The development and dissemination of a policy brief of the study findings will involve patients.

RESULT

Respondents' Profile

All pregnant women recruited under the two arms of the study consented to be observed. Under the quantitative arm, we observed 393 pregnant women. Most (54%) were between 15 and 24 years old with a median age of 23 years. A quarter were below the age of 20, half below the age of 23, and three quarters below the age of 30. The childbirths were through spontaneous vaginal delivery. Twin delivery occurred in only six (1.5%) cases. Also, 39% of the new mothers did not breastfeed their newborns in the first hour after delivery. Thirty-three health workers attended the deliveries under this arm of the study. The number of deliveries attended by each health worker ranged from one to thirty-seven, with an average of 12 (S.D: 10) deliveries each and a median of eight during the observation period. At least two health workers attended about 61% of the 393 deliveries. Also, Junior Community Health Extension Workers (JCHEWs) attended the majority (36%) of the 393 deliveries (Table 1).

Table 1. Pregnant women's profile

For peer review only

	Quantitative arm % n=393	Qualitative arms % n=27
Age		
<15	0	3
15 - 24	53	63
25 - 34	36	30
35+	11	4
Gestational age in weeks		
Mean	41 (SD=12)	38(SD=0.6)
Mode	38	38
Median	38	38
Parity		
Nulliparous (first pregnancy above 28wks gestational age (GA))	20	33
Multiparous (more than first pregnancy above 28wk GA)	80	67
Initiated breastfeeding within the first hour after birth		
no	39	37
yes	61	63
Health worker who attended pregnant woman's labor and delivery		
nurse/midwife	4	0
junior community health extension worker	36	28
community health extension worker	18	15
environmental health assistant/technician/officer	28	19
hospital assistant	10	19
nutritionists/dieticians/students	4	19
Sex of health worker that attended pregnant woman's labor and delivery		
male	1	0
female	99	100

Under the qualitative arm, 27 pregnant women were delivered of their babies by 16 health workers. The pregnant women consented to be observed and interviewed. The 16 health workers also consented to be interviewed after the observation. Most (63%) of the pregnant women were between 15 and 24 years old, with a median age of 22 years. A quarter were below 20 years of age and a quarter above 30 years of age. None had a twin delivery. Also, 37% of the mothers did not breastfeed the newborn within one hour of birth under this arm of the study. We also

interviewed all 27 mothers and 16 health workers one hour after childbirth. Of the 16 health care workers interviewed under the qualitative arm, most (63%) were hospital assistants. About a third (33%) were Community Health Extension Workers (CHEWS), 17% (2) were students, 6% (1) were nutritionist, and 6% (1) environmental health technician. Of the 27 deliveries under this arm of the study, in 44% of cases, the attending health care worker was assisted by another health worker (Table 1).

Knowledge of time to initiate breastfeeding

The qualitative arm shows that health workers know when mothers should breastfeed newborns. Ninety-two percent of them responded that breastfeeding should start between zero to sixty minutes (Table 2).

Table 2. Health workers' response to when breastfeeding should start.

**Attending health care workers interviewed under qualitative arm**	**Frequency**	**Percent** *(n=16)*
Response to when breastfeeding should start		
Immediately after delivery	9	54
**10 - 30 minutes after delivery**	2	13
**30 - 60 minutes after delivery**	1	8
**0 - 60 minutes after delivery**	3	17
**0 - 24 hours after delivery**	1	8

They attributed their knowledge to on-the-job training sessions on newborn care had with visiting clinical-mentors. They believe their knowledge is sustained via peer-to-peer discussions while on the job and during staff meetings. One Junior Community Health Extension Worker (JCHEW) said:

"breast feeding should commence immediately after birth …we have clinical mentors that visit and remind us of these things. We also have staff meetings where we remind ourselves of these practices" (birth attendant 1)

The word "immediately" is more used by lower cadre health workers to describe when breastfeeding should start. The CHEWs are more specific about when breastfeeding should start. They also link the time breastfeeding should start with its benefits. The quote below is a typical response from a Senior CHEW when she was asked when breastfeeding should start.

For peer review only

"It is very important because it helps the child to suck the yellowish nutrient in the breast milk. It boosts the child's immunity. It also helps the mother's uterus to shrink and close …helping to stop bleeding. It should start by 30min to 1hour after delivery" (birth attendant 2).

Barriers to early breastfeeding

Birth attendants' unwillingness or inability to accommodate mothers' safe traditional practices

The quantitative study shows that pregnant women denied safe traditional birth practices such as praying or reading religious texts during the second and third stages of labor are five times more likely not to breastfeed within the first hour (RR=4.5, 95% CI=1.2-17.1) compared to pregnant women allowed these practices (Table 3).

Table 3: Association between early breastfeeding and predictor variables in the study

Variable	Response	Women who didn't initiate breastfeeding within 1hr after birth		
		% n=154	*Relative risk 95% CI*	*p value*
birth attendant had received training on newborn care	No	49	1.2 (0.9-1.5)	0.2
	Yes	51		
there were delays in providing care	No	90	0.7 (0.5-1.03)	0.2
	Yes	10		
Communication was easy and frequent between woman and birth attendant	No	2	0.7 (0.3-1.8)	0.5
	Yes	98		
Poor staff attitude	No	96	0.7 (0.4-1.1)	0.2
	Yes	4		
woman denied some safe traditional childbirth practices*	No	99	4.5 (1.2-17.1)	0.003***
	Yes	1		
birth attendant determined the birth position	No	41	1 (0.8-1.3)	1
	Yes	59		
woman was allowed to give birth in the position she preferred**	No	37	0.9 (0.7-1.2)	0.5
	Yes	63		
the labor room was clean and comfortable	No	18	0.98 (0.7-1.4)	0.9
	Yes	82		

**n=154, **n=150, ***Fishers X^2*

When mother's state of health after childbirth isn't an issue, a typical response given by some mothers for not breastfeeding in the first hour was the need to first wash the breast or have a bath to feel clean.

"he (the baby) has to exercise patience until we get home (before he is breastfed). I can't breastfeed him before I take my bath" (mother 1)

The quantitative study suggests that the mother's need to be clean may not be influenced by the cleanliness of the environment. Initiating breastfeeding is not related to the cleanliness and comfort provided by the delivery room. Mothers who had their babies when the labor room was clean and comfortable were just as likely not to initiate breastfeeding early as those who had their babies when the labor room was not (RR=0.98; 95% CI 0.7-1.4; p=0.9).

Poor management of mothers' post-delivery state of health

Post-delivery pains and fatigue are barriers to breastfeeding within the first hour after birth. Even when mothers show a good knowledge of when breastfeeding should start, some still express the need to regain strength and wellness first before they breastfeed the newborn.

"breastfeeding should commence immediately after birth… I did not commence it because I was feeling after pains. The health worker said she will bring the baby to suck. I told her to allow me to have some relief" ZA (mother 2)

The quantitative study shows that pregnant women not encouraged to consume fluids or food at least once during labor are twice as likely not to breastfeed within the first hour compared with those encouraged to do so (RR=2.1; 95% CI=1.5-3; p=0.001). There is no evidence from the study that blood loss greater than 500mls during labor and delivery influence early breastfeeding (RR=1.17; 95% CI=0.2-5.9; p=1).

Human resource shortages

The qualitative study shows that shortage of health workers in PHCs introduces a delay in carrying out newborn care activities. Sometimes, health workers have to attend to other ill patients when there is no one else to assist. When we asked some health workers why a mother under their care did not breastfeed early, a typical response given was:

For peer review only

““You see, if you have someone that will assist you, you will assign the person to carry the baby to the mother and initiate the breastfeeding, or weigh the child, or apply chlorhexidine to the baby’s cord or any other thing needed while you continue with the remaining work and management of others …but most of the time you are on duty alone. It is because we have shortage of manpower here in this facility” (birth attendant 3)

The human resource shortage also affects rooming-in in the PHCs. Placing the mother and newborn in the same room after delivery is rooming-in[52]. Sometimes, the health worker has to clean and make this room ready for the mother and newborn. When there is a shortage of staff, this delays the transfer of the mother and newborn to the rooming-in room, and affects early breastfeeding.

“I wanted to transfer the mother and baby to the postnatal ward… before she commences breastfeeding (of the newborn). The (lying-in) room is not set (for use yet). I have to clean and make the room so she is comfortable to commence breastfeeding” (birth attendant 4)

Ineffective rooming-in practices

When rooming-in happens, the quantitative study reveals that mothers who do not have skin-to skin contact with their newborns in the first hour after birth are twice as likely not to breastfeed early, compared to mothers who did (RR=2.3, 95% CI=1.8-2.8; $p<0.001$). Just keeping the mother and newborn in the same room (rooming-in) without skin-to-skin contact has no influence on early breastfeeding (RR=2.6; 95% CI=2.3-2.9; $p=0.059$).

Lack of privacy and proper visiting-hour policy in the PHCs

The PHCs have open rooming-in rooms that doesn’t guarantee privacy. There are also no defined visiting hours in the PHCs. The qualitative arm of the study shows that male and female relatives visit the new mother in the hospital after childbirth. During the visit, the relatives pray for the newborn baby and congratulate the mother. Some relatives sit around after prayers for long. When relatives come visiting, the mothers have to dress up to receive them and do not breastfeed during this time. The birth attendants also delay supporting the mother to breastfeed. One birth attendant suggested that addressing the issue puts them in bad light in the community.

“you know, the people in the community have a unique character or attitude. The moment you try to talk to them about this kind of issue they feel you are molesting them or depriving them of

For peer review only

coming close to their relatives. They do not know you are trying to ensure their relative (mother and baby) gets what is beneficial to them" (birth attendant 5).

Facilitators of early breastfeeding

Health education during ANC and post-delivery period

From the qualitative study, we find that Antenatal Clinics (ANC) helps pregnant women to learn about breastfeeding newborns within one hour of birth. The knowledge they gain during these clinics influences their behavior after childbirth. Most mothers who practiced early breastfeeding said what they learned from ANC influenced their decision to do so. The typical response they gave is:

"I used to give my children water (after childbirth). I did not know the importance of breastfeeding early. I used to think breast feeding could start at any time of the day (of birth). I started breastfeeding early because the health workers tell us (of the importance of starting breastfeeding immediately after birth) during ANC" (mother 3)

Encouraging and supporting mothers to start breastfeeding after childbirth

From the qualitative study, we find that when the birth attendants encourage some reluctant mothers to breastfeed their newborns within one hour of childbirth; they do.

"I was told (by the health worker) to give (the baby breast to suck early enough). Normally, I won't" (mother 4)

The encouragement process takes the form of a negotiation between the birth attendant and the mother. We find that the information passed by the birth attendants to the mothers around the benefits of early breastfeeding, facilitate early breastfeeding in the PHCs.

"I commenced breastfeeding early because of what the health worker said …she explained how it is important to the health of my baby. That's why I commenced it" (mother 5)

Sometimes, showing the mothers how to place and breastfeed the newborns also facilitates early breastfeeding after childbirth in these health facilities.

"I gently encourage them and tell them to give (breast milk) early… I put the baby on her laps and remove the breast, and demonstrate to her how to breastfeed" (birth attendant 6)

For peer review only

DISCUSSION

In our study setting, we find that close to four out of every 10 newborns do not get breastfed within the first hour of birth. This doubles their risk of dying in the first 28 days of their lives[10,15]. Our estimate is four percentage points lower than what researcher observed in PHCs in Asia[31]. It is also higher than what others have estimated amongst mothers who had spontaneous vaginal deliveries (SVD) in some secondary health facilities; and lower than what others have estimated amongst mothers who had SVD in some tertiary health facilities, even in Nigeria[31,53,54]. The mixed results emphasize the influence of context on the early breastfeeding of newborns in different health facilities. It underscores the need for unique interventions to address the problem.

The northeast region of Nigeria has a shortage of skilled health workers. The Boko-Haram insurgency has made this worse[55–57]. It is also worsened by staff absenteeism in PHCs in the region. Only about 35% of employed staff in PHCs in Gombe are likely to be at work on any given day, for example[22]. Studies suggest that a shortage of human resource and a dominant population of unskilled health care workers affects the quality of newborn care in health facilities[17,58–60]. This is not overall consistent with our study findings. We find that unskilled health workers are dominant in our study setting. Also, we find that human resource shortage is a barrier to early initiation of breastfeeding in the PHCs. We did not find that the skills of the birth attendants influenced the early breastfeeding of newborns. This may be because clinical mentors have trained the birth attendants in our study on newborn care. Educational interventions around support for the breastfeeding of newborns has been found to improve health workers' knowledge, attitude, and compliance with the practice[61,62].

In our study, we find that the mothers denied safe traditional birth practices like praying, reciting religious texts, or reading religious books during deliveries were five times more likely not to breastfeed the newborns than the mothers not denied. This supports the findings from other settings that shows that the unwillingness of birth attendants to accommodate safe traditional birth practices affects mothers' adoption of supportive care[59,63,64]. This may be because mothers perceive denial of such traditional practices as mistreatment or abuse[59,65]. A study in Norway finds that the recent abuse of women by "both known and unknown" perpetrators affects their breastfeeding behavior[66]. Although in our study, we found no evidence to support this. Instead we find that birth attendants' attitude, rapport skills, and negligence during the delivery period doesn't affect early breastfeeding practice amongst mothers. The discrepancy in findings may be

because the women in our study setting perceive or tolerate abuse differently than women in other settings[67].

Washing of the breast with water after childbirth is a hygiene related practiced in parts of the world[68]. In our study, mothers express a strong need to wash the breast and/or have a bath before breastfeeding newborns. This makes the “need for a bath after childbirth” a major theme for why mothers delay breastfeeding newborns in our study setting. Some health workers also agree that mothers should wash their breasts first before breastfeeding newborns after childbirth[18]. Their inability to help the mothers to wash their breasts or have a bath before breastfeeding may be because there is no running water in the health facility. Only 38% of priority PHCs in Gombe have running water[22]. The need to wash the breast may be deeper than the need for hygiene, nonetheless. Washing of the breast or having a bath after childbirth before breastfeeding newborns has traditional and/or religious undertones in other parts of the world[60,69].

The other barriers that influence early breastfeeding of newborn in PHCs that our study finds are post-delivery pains and fatigue within the first hour after birth, delay in skin to skin contact during rooming-in, and poor visiting hour policy in the PHC. These findings reinforces what other studies have reported[5,21,24,33,41,54,60]. The post-delivery fatigue may be because the mother is famished. This may also be due to anemia, infections, thyroid disorders, mood disorders and cardiomyopathy[70] which our study does not assess. The quantitative arm of our study shows that mothers not encouraged to take fluids or eat during the second and third stages of labor are more likely not to breastfeed early after childbirth. There is no evidence from our study that the fatigue may be due to blood loss. We also find that rooming-in is not enough and that skin to skin contact must be deliberate. Mothers that did not have skin to skin contact with their newborns while in the rooming-in room were more likely not to breastfeed than those who did in our study. Our study reemphasizes that the lack of restrictions on relatives visiting the mothers in the post-delivery period impedes breastfeeding[71]. This could be because the mothers don’t feel comfortable breastfeeding in front of male relatives or visitors.

Helping mothers to breastfeed within an hour of birth is an international recommendation practiced by health care providers in most of the world[32,72]. We find that birth attendants in our study setting practice this and the strategy facilitates the early breastfeeding of newborns in public PHCs. This may be because there is an awareness of the recommendation[73]. Our study also reinforces findings from previous studies on the benefits of health education on the timely

For peer review only - http://bmjopen.bmj.com/site/about/guidelines.xhtml

breastfeeding of newborns[74,75]. In our study, we find that health education received by mothers during ANC and the post-delivery period improves the practice of early initiation of breastfeeding in public PHCs, in our study setting

CONCLUSION

Only about 60% of babies born in public PHCs in Northeast Nigeria get breastfed in the first hour of birth. This means the rest miss important nutrition that saves lives. The stakeholders in the region must increase their focus on improving the breastfeeding practices in public PHCs. This will improve the survival of newborns and impact of their investments. Instituting policies that protect mothers' privacy; and finding innovative ways to accommodate and promote safe traditional practices in the intrapartum and postpartum period in PHCs will improve the early breastfeeding of newborns in these PHCs. Birth attendants in PHCs must also be trained on effective rooming-in to further improve early breastfeeding of newborns in these public PHCs, however.

LIST OF ABBREVIATIONS

PHC (Primary Health Care hospital/facility), ANC (Antenatal Care) VHW (Village Health Workers), CHEWs (Community Health Extension Workers).

DECLARATIONS

Ethics approval and consent to participate

The Gombe State Ministry of Health Ethics Committee granted ethical approval for the qualitative arm of the study (reference no: MOH/ADM/658/VOL.II/104). Mothers and birth attendants also gave their consent to take part in the study before the interviews. The IDEAS team at the London School of Hygiene and Tropical Medicine (LSHTM) got ethical approval for the quantitative arm of the study from LSHTM (reference 6088).

Funding

The study is funded as part of the evaluation of SFH's MNCH2 project being funded by the Bill and Melinda Gates Foundation and implemented in Gombe State. The views and opinions expressed in the paper are those of the authors and do not reflect those of SFH and/or GSPHCDA. The

For peer review only

intervention is funded through donor grants received by SFH. The donor did not play a role in the design of this evaluation.

Data Availability

Data for the quantitative arm of the study is in a secured database at the London School of Hygiene and Tropical Medicine. Write to Nasir Umar through nasir.umar@lshtm.ac.uk to place a reasonable request for the anonymized version of it. Also, write to Shobo Olukolade, through shoboolukolade@gmail.com to ask for the anonymized transcripts of the qualitative data.

Competing interest

OGS and PL are consultants working for Society for Family Health (SFH), OI and JA are full-time staff of SFH. GA is the Former Executive Secretary of Gombe State Primary Health Care Development Agency. SFH's program in Northeast Nigeria seeks to improve MNCH outcomes in the general population.

Author Contribution

OGS conceived of the study and developed the original draft of the manuscript. OGS analyzed the quantitative data. OGS and PL analyzed the qualitative data. NU, GA, JA, and OI reviewed, edited, and made significant contributions to the development of the final manuscript. All authors read and approved the final manuscript.

Acknowledgement

We thank all the staff of SFH MNCH2 project in Gombe, particularly Mrs. Magdalene Okolo for their contributions to the study. We also thank the field staff who conducted the observations and interviews. We also thank our partners, the members of the IDEAS team from the London

For peer review only

School of Hygiene and Tropical Medicine, for leadership in designing and implementing the quantitative arm of the study.

REFERENCES

1. World Health Organization. *Reaching the every newborn national 2020 milestones: country progress, plans and moving forward*. http://apps.who.int/iris/bitstream/handle/10665/255719/9789241512619-eng.pdf?sequence=1 (2017).
2. Edmond, K. M. *et al.* Delayed Breastfeeding Initiation Increases Risk of Neonatal Mortality. *Pediatrics* **117**, e380–e386 (2006).
3. Mullany, L. C. *et al.* Breast-Feeding Patterns, Time to Initiation, and Mortality Risk among Newborns in Southern Nepal. *J Nutr* **138**, 599–603 (2008).
4. World Health Organization. *Every newborn: an action plan to end preventable deaths*. (World Health Organization, 2014).
5. WHO | Early initiation of breastfeeding to promote exclusive breastfeeding. *WHO* http://www.who.int/elena/titles/early_breastfeeding/en/.
6. WHO | Newborn care at birth. *WHO* http://www.who.int/maternal_child_adolescent/newborns/care_at_birth/en/.
7. Debes, A. K., Kohli, A., Walker, N., Edmond, K. & Mullany, L. C. Time to initiation of breastfeeding and neonatal mortality and morbidity: a systematic review. *BMC Public Health* **13**, S19 (2013).
8. UNICEF & WHO. *Capture the moment: early initiation of breastfeeding : the best start for every newborn*. (2018).
9. Friedrich, M. J. Early Initiation of Breastfeeding. *JAMA* **320**, 1097–1097 (2018).

For peer review only

10. al, K. J., et. Timing of breastfeeding initiation and exclusivity of breastfeeding during the first month of life: effects on neonatal mortality and morbidity--a ... - PubMed - NCBI. https://www.ncbi.nlm.nih.gov/pubmed/24894730.

11. Federal Ministry of Health. Nigeria Every Newborn Action Plan: A plan to end preventable newborn deaths in Nigeria. (2016).

12. Anyamele, O., Akanegbu, B., Assad, J.-C. & Ukawuilulu, J. Differentials in Infant and Child Mortality in Nigeria: Evidence from Pooled 2003 and 2008 DHS Data. *Advances in Management and Applied Economics* **7**, 73–96 (2017).

13. Morakinyo, O. M. & Fagbamigbe, A. F. Neonatal, infant and under-five mortalities in Nigeria: An examination of trends and drivers (2003-2013). *PLOS ONE* **12**, e0182990 (2017).

14. Tura, G., Fantahun, M. & Worku, A. The effect of health facility delivery on neonatal mortality: systematic review and meta-analysis. *BMC Pregnancy Childbirth* **13**, 18 (2013).

15. Khan, J., Vesel, L., Bahl, R. & Martines, J. C. Timing of Breastfeeding Initiation and Exclusivity of Breastfeeding During the First Month of Life: Effects on Neonatal Mortality and Morbidity—A Systematic Review and Meta-analysis. *Matern Child Health J* **19**, 468–479 (2015).

16. Takahashi, K. *et al.* Prevalence of early initiation of breastfeeding and determinants of delayed initiation of breastfeeding: secondary analysis of the WHO Global Survey. *Scientific Reports* **7**, 44868 (2017).

17. Haile, Z. T. *et al.* The Moderating Effect of Urban Versus Rural Residence on the Relationship Between Type of Birth Attendant and Early Initiation of Breastfeeding in Ghana. *J Hum Lact* **34**, 810–820 (2018).

For peer review only

18. Bee, M., Shiroor, A. & Hill, Z. Neonatal care practices in sub-Saharan Africa: a systematic review of quantitative and qualitative data. *Journal of Health, Population and Nutrition* **37**, 9 (2018).

19. Sobel, H. L., Silvestre, M. A. A., Iii, J. B. V. M., Oliveros, Y. E. & Nyunt-U, S. Immediate newborn care practices delay thermoregulation and breastfeeding initiation. *Acta Paediatrica* **100**, 1127–1133 (2011).

20. Adewuyi, E. O., Zhao, Y., Khanal, V., Auta, A. & Bulndi, L. B. Rural-urban differences on the rates and factors associated with early initiation of breastfeeding in Nigeria: further analysis of the Nigeria demographic and health survey, 2013. *International Breastfeeding Journal* **12**, 51 (2017).

21. Berde, A. S. & Yalcin, S. S. Determinants of early initiation of breastfeeding in Nigeria: a population-based study using the 2013 demograhic and health survey data. *BMC Pregnancy and Childbirth* **16**, 32 (2016).

22. Marchant, T. *Change in maternal and newborn health care interactions between families and frontline workers. Report from Gombe State, Nigeria, 2012-2015*. https://ideas.lshtm.ac.uk/wp-content/uploads/2017/08/Marchant_IDEAS_Gombe_State_Follow-upSurvey_2016.pdf (2016).

23. Thulier, D. & Mercer, J. Variables Associated With Breastfeeding Duration. *Journal of Obstetric, Gynecologic & Neonatal Nursing* **38**, 259–268 (2009).

24. Ahmed, A. E. & Salih, O. A. Determinants of the early initiation of breastfeeding in the Kingdom of Saudi Arabia. *International Breastfeeding Journal* **14**, 13 (2019).

For peer review only

25. Liben, M. L. & Yesuf, E. M. Determinants of early initiation of breastfeeding in Amibara district, Northeastern Ethiopia: a community based cross-sectional study. *International Breastfeeding Journal* **11**, 7 (2016).

26. Delivery care. *UNICEF DATA* https://data.unicef.org/topic/maternal-health/delivery-care/.

27. Bohren, M. A. *et al.* Facilitators and barriers to facility-based delivery in low- and middle-income countries: a qualitative evidence synthesis. *Reproductive Health* **11**, 71 (2014).

28. Campbell, O. M. & Graham, W. J. Strategies for reducing maternal mortality: getting on with what works. *The Lancet* **368**, 1284–1299 (2006).

29. WHO | Early initiation of breastfeeding. *WHO* http://www.who.int/elena/titles/commentary/early_breastfeeding/en/.

30. Forster, D. A. & McLachlan, H. L. Breastfeeding Initiation and Birth Setting Practices: A Review of the Literature. *Journal of Midwifery & Women's Health* **52**, 273–280 (2007).

31. Karim, F. *et al.* Initiation of breastfeeding within one hour of birth and its determinants among normal vaginal deliveries at primary and secondary health facilities in Bangladesh: A case-observation study. *PLOS ONE* **13**, e0202508 (2018).

32. WHO | Protecting, promoting and supporting breastfeeding in facilities providing maternity and newborn services. *WHO* http://www.who.int/elena/titles/full_recommendations/breastfeeding-support/en/.

33. Sharma, I. K. & Byrne, A. Early initiation of breastfeeding: a systematic literature review of factors and barriers in South Asia. *International Breastfeeding Journal* **11**, 17 (2016).

34. DiGirolamo, A. M., Grummer-Strawn, L. M. & Fein, S. Maternity Care Practices: Implications for Breastfeeding. *Birth* **28**, 94–100 (2001).

For peer review only

35. Fedreal Ministry of Health. Nigeria Investment case for reproductive, maternal, newborn, child, adolescent health and nutrition: 2017 - 2030. (2017).

36. The Maternal and Neonatal Health Project in North East Nigeria 2012 – 2016 | Society for Family Health, Nigeria. http://www.sfhnigeria.org/the-maternal-and-neonatal-health-project-in-north-east-nigeria-2012-2016/.

37. State Accountability and Quality Improvement Project. *Pact* https://www.pactworld.org/country/nigeria/project.

38. Strengthening the healthcare system in north-east Nigeria - a priority for WHO | WHO | Regional Office for Africa. https://www.afro.who.int/news/strengthening-healthcare-system-north-east-nigeria-priority-who.

39. Makinde, O. A., Sule, A., Ayankogbe, O. & Boone, D. Distribution of health facilities in Nigeria: Implications and options for Universal Health Coverage. *The International Journal of Health Planning and Management* https://onlinelibrary.wiley.com/doi/abs/10.1002/hpm.2603 (2018) doi:10.1002/hpm.2603.

40. Nigeria Health Facility Registry. https://hfr.health.gov.ng/facilities/search?_token=r2vSeMamYxcYqkwMtdRF67CgGTWkRNkpYS6QoUQV&facility_type_id=1&state_id=115&lga_id=&ward_id=0&facility_level_id=2&ownership_id=1&operational_status_id=0&registration_status_id=0&license_status_id=0&geo_codes=0&service_type=0&service_category_id=0&facility_name=.

41. Bbaale, E. Determinants of Early Initiation, Exclusiveness, and Duration of Breastfeeding in Uganda. *J Health Popul Nutr* **32**, 249–260 (2014).

42. Li, R., Scanlon, K. S. & Serdula, M. K. The Validity and Reliability of Maternal Recall of Breastfeeding Practice. *Nutr Rev* **63**, 103–110 (2005).

For peer review only

43. Kawulich, B. B. Participant Observation as a Data Collection Method. *Forum Qualitative Sozialforschung / Forum: Qualitative Social Research* **6**, (2005).

44. Altmann, J. Observational Study of Behavior: Sampling Methods. *Behaviour* **49**, 227–267 (1974).

45. Schoonenboom, J. & Johnson, R. B. How to Construct a Mixed Methods Research Design. *Kolner Z Soz Sozpsychol* **69**, 107–131 (2017).

46. Denzin, N. K. & Lincoln, Y. S. *The SAGE Handbook of Qualitative Research*. (SAGE, 2011).

47. Gombe State Government. Gombe State Nigeria: History. *Gombe State* http://gombestate.gov.ng/history-2/.

48. Federal Ministry of Health. Federal Republic of Nigeria Draft National Human Resources for Health Strategic Plan 2008 - 2012. (2012).

49. Alenoghena, I. O., Isah, E. C. & Isara, A. R. Availability and Type of Human Resource for Health in Public Primary Health Care Facilities in Selected Communities, Edo State. *Journal of Community Medicine and Primary Health Care* **28**, 11–18 (2016).

50. Marchant, D. T. Maternal and newborn health care. Baseline findings from Gombe State, Nigeria. https://ideas.lshtm.ac.uk/report/mnh-care-baseline-gombe-state/ (2013) doi:10.17037/PUBS.01126638.

51. Patton, M. Designing Qualitative Studies. in *Qualitative evaluation and research methods* 169–186 (SAGE, 1990).

52. Jaafar, S. H., Ho, J. J. & Lee, K. S. Rooming-in for new mother and infant versus separate care for increasing the duration of breastfeeding. *Cochrane Database Syst Rev* CD006641–CD006641 (2016) doi:10.1002/14651858.CD006641.pub3.

53. Örün, E. *et al.* Factors associated with breastfeeding initiation time in a Baby-Friendly Hospital. *The Turkish Journal of Pediatrics* **52**, 10–16 (2010).

For peer review only

54. Awi, D. D. & Alikor, E. a. D. Barriers to timely initiation of breastfeeding among mothers of healthy full-term babies who deliver at the University of Port Harcourt Teaching Hospital. *Nigerian Journal of Clinical Practice* **9**, 57-64–64 (2006).

55. Federal Ministry of Health. National Human Resource for Health Strategic Plan: 2008 - 2012. (2008).

56. Premium Times Nigeria. Gombe govt. waives retirement for 100 nurses, midwives due to manpower shortage. https://www.premiumtimesng.com/news/more-news/230165-gombe-govt-waives-retirement-100-nurses-midwives-due-manpower-shortage.html (2017).

57. Omoluabi, E. *Needs Assessment of the Nigerian Health Sector*. https://nigeria.iom.int/sites/default/files/newsletter/ANNEX%20XXIV%20Needs%20Assessment%20of%20the%20Nigeria%20health%20Sector.pdf (2014).

58. Aluko, J. O., Anthea, R. & Marie Modeste, R. R. Manpower capacity and reasons for staff shortage in primary health care maternity centres in Nigeria: a mixed-methods study. *BMC Health Services Research* **19**, 10 (2019).

59. Bohren, M. A. *et al.* The Mistreatment of Women during Childbirth in Health Facilities Globally: A Mixed-Methods Systematic Review. *PLoS Med* **12**, e1001847 (2015).

60. Majra, J. P. & Silan, V. K. Barriers to Early Initiation and Continuation of Breastfeeding in a Tertiary care Institute of Haryana: A Qualitative Study in Nursing Care Providers. *J Clin Diagn Res* **10**, LC16–LC20 (2016).

61. Balogun, O. O. *et al.* Health Facility Staff Training for Improving Breastfeeding Outcome: A Systematic Review for Step 2 of the Baby-Friendly Hospital Initiative. *Breastfeeding Medicine* **12**, 537–546 (2017).

For peer review only

62. Okolo, S. N. & Ogbonna, C. Knowledge, attitude and practice of health workers in Keffi local government hospitals regarding Baby-Friendly Hospital Initiative (BFHI) practices. *Eur J Clin Nutr* **56**, 438–441 (2002).

63. Mannava, P., Durrant, K., Fisher, J., Chersich, M. & Luchters, S. Attitudes and behaviours of maternal health care providers in interactions with clients: a systematic review. *Globalization and Health* **11**, 36 (2015).

64. Chatters, L. M. Religion and Health: Public Health Research and Practice. *Annual Review of Public Health* **21**, 335–367 (2000).

65. Beck, C. T. A Secondary Analysis of Mistreatment of Women During Childbirth in Health Care Facilities. *Journal of Obstetric, Gynecologic & Neonatal Nursing* **47**, 94–104 (2018).

66. Sørbø, M. F., Lukasse, M., Brantsæter, A.-L. & Grimstad, H. Past and recent abuse is associated with early cessation of breast feeding: results from a large prospective cohort in Norway. *BMJ Open* **5**, e009240 (2015).

67. Maya, E. T. *et al.* Women's perspectives of mistreatment during childbirth at health facilities in Ghana: findings from a qualitative study. *Reproductive Health Matters* **26**, 70–87 (2018).

68. Khan, M. E. Shaping Demand and Practices to Improve Family Health Outcomes: Findings from a Quantitative Survey, Volume 3: Bihar. 221.

69. Tawiah-Agyemang, C., Kirkwood, B. R., Edmond, K., Bazzano, A. & Hill, Z. Early initiation of breast-feeding in Ghana: barriers and facilitators. *Journal of Perinatology* **28**, S46–S52 (2008).

70. Atkinson, L. S. & Baxley, E. G. Postpartum fatigue. *Am Fam Physician* **50**, 113–118 (1994).

For peer review only

71. Saxena, M., Srivastava, A., Dwivedi, P. & Bhattacharyya, S. Is quality of care during childbirth consistent from admission to discharge? A qualitative study of delivery care in Uttar Pradesh, India. *PLOS ONE* **13**, e0204607 (2018).

72. UNICEF & World Health Organization. Country Experiences with the Baby-Friendly Hospital Initiatiive. Compendium of Case Studies from Around the World. (2017).

73. Kakrani, V. A., Rathod (Waghela), H. K., Mammulwar, M. S. & Bhawalkar, J. S. Awareness about "Ten Steps for Successful Breastfeeding" among Medical and Nursing Students. *Int J Prev Med* **6**, (2015).

74. Guise, J.-M. *et al.* The Effectiveness of Primary Care-Based Interventions to Promote Breastfeeding: Systematic Evidence Review and Meta-Analysis for the US Preventive Services Task Force. *Ann Fam Med* **1**, 70–78 (2003).

75. de Oliveira, M. I. C., Camacho, L. A. B. & Tedstone, A. E. Extending Breastfeeding Duration Through Primary Care: A Systematic Review of Prenatal and Postnatal Interventions. *J Hum Lact* **17**, 326–343 (2001).

For peer review only

BMJ Open

Factors influencing the early initiation of breastfeeding in public primary health care facilities in Northeast Nigeria: A mixed methods study

Journal:	*BMJ Open*
Manuscript ID	bmjopen-2019-032835.R2
Article Type:	Research
Date Submitted by the Author:	02-Mar-2020
Complete List of Authors:	Shobo, Olukolade; Society for Family Health , Monitoring and Evaluation Department<br>Umar, Nasir; London School of Hygiene and Tropical Medicine Faculty of Infectious and Tropical Diseases, Disease Control<br>Gana, Ahmed; Gombe State Primary Health Care Development Agency, Office of the Executive Secretary<br>Longtoe, Peter; Society for Family Health , Monitoring and Evaluation Department<br>Idogho, Omokhudu; Society for Family Health , Office of the Managing Director<br>Anyanti, Jennifer; Society for Family Health , Office of the Deputy Managing Director
**Primary Subject Heading**:	Health services research
Secondary Subject Heading:	Nutrition and metabolism, Obstetrics and gynaecology
Keywords:	Health policy < HEALTH SERVICES ADMINISTRATION & MANAGEMENT, Quality in health care < HEALTH SERVICES ADMINISTRATION & MANAGEMENT, PRIMARY CARE, NEONATOLOGY

SCHOLARONE™ Manuscripts

For peer review only - http://bmjopen.bmj.com/site/about/guidelines.xhtml

I, the Submitting Author has the right to grant and does grant on behalf of all authors of the Work (as defined in the below author licence), an exclusive licence and/or a non-exclusive licence for contributions from authors who are: i) UK Crown employees; ii) where BMJ has agreed a CC-BY licence shall apply, and/or iii) in accordance with the terms applicable for US Federal Government officers or employees acting as part of their official duties; on a worldwide, perpetual, irrevocable, royalty-free basis to BMJ Publishing Group Ltd ("BMJ") its licensees and where the relevant Journal is co-owned by BMJ to the co-owners of the Journal, to publish the Work in this journal and any other BMJ products and to exploit all rights, as set out in our licence.

The Submitting Author accepts and understands that any supply made under these terms is made by BMJ to the Submitting Author unless you are acting as an employee on behalf of your employer or a postgraduate student of an affiliated institution which is paying any applicable article publishing charge ("APC") for Open Access articles. Where the Submitting Author wishes to make the Work available on an Open Access basis (and intends to pay the relevant APC), the terms of reuse of such Open Access shall be governed by a Creative Commons licence – details of these licences and which Creative Commons licence will apply to this Work are set out in our licence referred to above.

Other than as permitted in any relevant BMJ Author's Self Archiving Policies, I confirm this Work has not been accepted for publication elsewhere, is not being considered for publication elsewhere and does not duplicate material already published. I confirm all authors consent to publication of this Work and authorise the granting of this licence.

For peer review only

Factors influencing the early initiation of breastfeeding in public primary health care facilities in Northeast Nigeria: A mixed methods study

Corresponding Author:

1. Olukolade George Shobo (Society for Family Health, 8 Port Harcourt Crescent, Area 11, Garki, Abuja. Email: shoboolukolade@gmail.com)

Coauthors:

2. Nasir Umar (London School of Hygiene and Tropical Medicine, London. Email: nasir.umar@lshtm.ac.uk)
3. Gana Ahmed (Gombe State Primary Health Care Development Agency. Gombe State. Email: ahmedgana567@yahoo.com)
4. Peter Longtoe (Society for Family Health, 8 Port Harcourt Crescent, Area 11, Garki, Abuja. Email: plongtoe.com)
5. Omokhudu Idogho (Society for Family Health, 8 Port Harcourt Crescent, Area 11, Garki, Abuja. Email: oidogho@sfhnigeria.org)
6. Jennifer Anyanti (Society for Family Health, 8 Port Harcourt Crescent, Area 11, Garki, Abuja. Email: janyanti@sfhnigeria.org)

ABSTRACT

Introduction

The early initiation of breastfeeding is a high-impact intervention that gives newborns a better chance of survival. We assess the barriers and facilitators influencing the practice of early breastfeeding of newborns in public primary healthcare facilities (PHCs) in Northeast Nigeria, to influence the planning of programs targeted at improving newborn care in the region.

Method

We used an explanatory mixed methods approach. We conducted case-observation of childbirths and newborn care for the quantitative arm, and interviewed mothers and birth attendants one hour after childbirth for the qualitative arm. The analysis for the quantitative arm was done with SPSS version 23. For the qualitative arm, we transcribed the audio files, coded the texts, and categorized them using thematic analysis.

Result

We observed 393 and 27 mothers for the quantitative and qualitative arms of the study respectively. The quantitative arm shows that 39% of mothers did not breastfeed their newborns within one hour of birth. The qualitative arm shows that 37% of mothers did not breastfeed within one hour of birth. Themes that describe the barriers to early breastfeeding in public PHCs are:

For peer review only

birth attendants' unwillingness or inability to accommodate mothers' safe traditional practices, ineffective rooming-in practices, staff shortages, lack of privacy in the lying-in ward, and poor implementation of visiting-hour policy in public PHCs. The pregnant women denied safe traditional birth practices like chanting, praying, or reading religious books during delivery are five times more likely not to breastfeed newborns within the first hour of birth (RR=4.5, 95% CI=1.2-17.1) compared to pregnant women allowed these practices.

Conclusion

Stakeholders must increase their focus on improving the breastfeeding practices in the public PHCs. Instituting policies that protect mothers' privacy; and finding innovative ways to accommodate and promote safe traditional practices in the intrapartum and postpartum period in PHCs will improve the early breastfeeding of newborns in these PHCs.

STRENGHTS AND LIMITATIONS OF THE STUDY

- We used a mixed methods study design to provide a richer understanding of the factors that affect newborn breastfeeding practice in public PHCs in resource poor settings.
- We used purposive sampling techniques, focusing on information-rich public PHCs. This improved the chances of observing a high number of childbirths, and identifying typical implementation issues associated with the study aim, during the study period.
- Our study findings are not causal.
- The study findings may not generalize to secondary and tertiary health facilities, nor to PHCs located in places not similar to our study setting.

INTRODUCTION

Every year, about 77 million (50%) newborns do not get breastfed in the first hour of birth globally[1]. This leaves them vulnerable to diseases and death[1–3]. Newborn deaths continue to account for close to half of all under-5 mortalities across the world[4]. The early initiation of breastfeeding which means breastfeeding a newborn within one hour of birth[5], is a high-impact intervention[4,6,7] that gives newborns a better chance of survival[8]. It also provides them long-term health benefits[9,10]. The Northeast region of Nigeria has one of the highest newborn mortalities in

For peer review only

the world[11–13]. The early initiation of breastfeeding can reduce the risk of these newborn deaths by about a third[14,15].

The practice of early breastfeeding of newborns differs between and within countries[16,17]. For instance, it ranges between 17% to 95% for countries in sub-Saharan Africa [18,19]. In Nigeria, only about 35% of newborns get breastfed within the first hour after birth [20,21]. In the Northeast region of the country, only about 40% of mothers commence the breastfeeding of newborns in the first hour after childbirth[21]. In Gombe State, the estimate is 49%[22]. In the rural areas of the country, mothers are more likely not to practice it at all[20]. The mothers' age, level of education, and socioeconomic status are factors that influence the pattern of early initiation of breastfeeding in the general population. Others are maternal and newborn health problems, childbirth method, family support, availability of supplements, and maternal preference[21,23–25].

To improve early breastfeeding and avert newborn deaths, one approach is for more births to occur in health facilities[14,26–28]. Having a birth in a health facility improves the chance that a newborn will breastfeed early[21]. Evidence however suggests that poor birthing practices occur in health facilities which can disrupt the early start of breastfeeding[17,29–31]. While international recommendations for improving breastfeeding practices in health facilities exist[32], the problems faced by health facilities around the practice are context specific[33,34], requiring unique responses. Researches focusing on exploring and understanding these specific birthing practices in health facilities are now emerging[18,29].

The Northeast region of Nigeria is witnessing an increase in health investments by governments and non-governmental organizations[35–38]; aimed at reducing maternal and newborn deaths through improved access to quality childbirth and newborn care services in primary health care hospitals (PHCs)[35,36]. In Gombe State in Northeast Nigeria for instance, the government is implementing a Village Health Worker program that improves access to quality obstetric and newborn care services in public PHCs, through community-based demand generation activities. Primary health care facilities are better positioned to deliver high-impact newborn interventions in Nigeria. They make up 88% of health facilities in the country. In the Northeast, there are 5,086 public PHCs, and they make up 87% of the health facilities in the region[39,40]. Understanding the factors that influence the quality and uptake of life-saving newborn care services in these public PHCs is important for improving the effectiveness of the health investments in the region.

For peer review only

In this study, we assess the barriers and facilitators influencing early breastfeeding of newborns in public PHCs in Northeast Nigeria. Most studies assessing breastfeeding practice in health facilities have used secondary data from demographic health surveys[21,24,25,41]. While maternal recall is valid and reliable in establishing the period of initiation of breastfeeding[42], the DHS findings do not provide context concerning supply side factors that influenced the maternal behavior. A recent quantitative study in Bangladesh shows that about 43% of mothers in PHCs in the country do not breastfeed their newborns within one hour of birth. It did not assess the contextual implementation issues that influence the practice[31]. The literature around the contextual issues affecting the practice in PHCs in Africa is also lacking. Our approach uses a broader study design that helps to develop a deeper understanding of early breastfeeding practices in public PHCs in Northeast Nigeria[43,44]. We believe our findings will contribute to the discussions about health investments and strategies for improving newborn care in the region, and settings similar to it.

METHOD

Study Design

We used an explanatory mixed methods approach for this study. Mixed methods study designs deepen how we understand a research phenomenom[45,46]. In the explanatory mixed method type, a first phase quantitative data collection and analysis is followed by the collection of qualitative data, to explain the quantitative result[45]. The mixed-method approach helps us better understand the factors that influence the early initiation of breastfeeding in public PHCs[45]. We conducted the quantitative arm over four weeks in December 2017, and the qualitative arm over one week in November 2018. Budget constraints delayed implementing the qualitative arm. We assume that the time difference between the study arms is not sufficient to change the practice around breastfeeding newborns in the study setting.

Study Setting

We conducted the study in Gombe State, in the center of Northeast Nigeria on latitude 9" 30' and 12" 30'N, Longitude 8" 5'and 11" 45'E. It borders Borno, Yobe, Adamawa, Taraba, and Bauchi State. It has 11 Local Government Areas (LGAs) and 114 political wards[47]. There are 603 health facilities across the 11 LGAs in the State, 530 of which are public PHCs[40]. Of the 530 public PHCs in the State, the government has designated 114 as priority PHCs. These 114 are Ward Health

For peer review only - http://bmjopen.bmj.com/site/about/guidelines.xhtml

Centers and provide basic emergency obstetric and newborn care services. Nurses, community health workers, community health extension workers (CHEWs), junior CHEWs, and environmental health officers are the main staff of PHCs in Nigeria[48]. Community Health Officer, a Public Health Nurse, three CHEWs, four Nurse/Midwives and one medical Assistant are the main staff of a Ward Health Center[49].

Fifty percent of these priority PHCs have staff trained in providing basic emergency obstetric and newborn care services. About 36% of them have labor rooms and lying-in wards. None has a medical doctor, 4% have at least one nurse, and 19% have at least one midwife. Each PHC has an average of six health workers not categorized as skilled in attending labor and delivery. These include Community Health Extension Workers (CHEWs), environmental health technicians, hospital assistants (cleaners), and students. The majority (34%) of the staff in these PHCs have no medical training[50]. Fewer than half of pregnant women in the State access pregnancy care at least four times as recommended, and only about a third access facility based intra-partum care and/or skilled attendance at birth[50]. Cesarean sections are not conducted in PHCs in the study setting.

PHC Selection

Quantitative arm: We selected 10 of the 114 priority PHCs using purposive sampling technique. The 10 had the most deliveries per day on the average in the six-month period prior to starting the study, thus suited to provide rich information on our study objective. They also had labor wards and lying-in wards. The budget for the study dictated the number of health facilities chosen.

Qualitative arm: We selected three of the 10 priority PHC in the quantitative arm of the study. The three had the most deliveries per day on the average. They also had labor wards and lying-in wards. The intensity of the childbirths in the three PHCs and over the study period, will illuminate the barriers and facilitators of early initiation of breastfeeding in public PHCs in the region[51].

Subject Selection

We included all mothers who delivered in the selected PHCs and gave their consent to take part in the study in both arms of the study. For the qualitative arm: we also recruited all consenting healthcare providers who attended deliveries; and the mothers with a live birth for interviews.

Instrument and data collection

Quantitative arm: We recruited and trained nurses and nurse-midwives who are not a part of the health facility staff to complete the assessment tool. They observed and documented the time of events from when a pregnant woman in labor entered the health facility to when she leaves after childbirth or referral. During data collection, they only observed and did not take part or comment in the care offered to the pregnant woman. We only told them to intervene or offer help during a life-threatening situation to the mother and/or baby. The data collectors used separate assessment tools in cases of twin deliveries.

The health facility staff and pregnant women knew of the nurse and nurse-midwives but were unaware of what aspect of care was being observed. Data on the cadre of the health worker, events during the first to third stage of labor, and newborn care activities in the first hour after birth were collected. The nurses and nurse-midwives were available for 24hours in each of the health facilities throughout the study period on a shift schedule. They observed all deliveries.

Qualitative arm: We also recruited and trained female nurses and midwives who are not part of the health facility staff to observe and complete the assessment tool under this arm of the study. Observation for each pregnant woman started during the second stage of labor and ended one hour after childbirth under this arm of the study. The data collector then interviewed the mother and attendant health worker, after the observation period. The assessment tool used for the observation is an extract from the tool used for the quantitative arm. For the mothers and birth attendants we asked questions around how they felt about the childbirth process and knowledge of when newborn babies should breastfeed during the interview. We also asked the mothers why they chose to or not to breastfeed their newborns within the hour depending on if they did or not. Also, we asked the attending healthcare workers why they think breastfeeding occurred or did not occur within the first hour for each newborn. We also asked the attending healthcare workers about the strategies they used to encourage mothers to breastfeed their newborns within the hour. The data collectors attended every birth in the health facilities and interviewed respondents who could not speak or understand English in Hausa. They interviewed the mother and attending healthcare worker separately. The mothers' interviews occurred at their bedside in the lying-in ward. The data collectors excused relatives or visitors in the lying-in room during interviews.

Analysis

For peer review only

Quantitative arm: We analyzed the data using SPSS version 23. We present nominal variables as percentages. We also determined associations and relative risks between initiation of breastfeeding and predictor categorical variables using two-by-two contingency tables. To assess significant associations, we used Fisher's or Pearson's Chi-Squared test as appropriate

Qualitative arm: We transcribed the interviews from their audio files and analyzed the data with Saturate, an online qualitative software. Two people on the team analyzed a subset of the data and generated codes. The two people then came together to review and agree on the codes generated and their meaning, eliminating less useful codes. The two reviewers held regular face-to-face meetings to discuss their codes. We then analyzed the rest of the dataset using the agreed code-framework developed by the two. We generated themes from the codes using an inductive approach.

Patient and Public Involvement Statement

Resources for patient and public involvement statement was unavailable, so we could not involve patients. The development and dissemination of a policy brief of the study findings will involve patients.

RESULT

Respondents' Profile

All pregnant women recruited under the two arms of the study consented to be observed. Under the quantitative arm, we observed 393 pregnant women. Most (54%) were between 15 and 24 years old with a median age of 23 years. A quarter were below the age of 20, half below the age of 23, and three quarters below the age of 30. The childbirths were through spontaneous vaginal delivery. Twin delivery occurred in only six (1.5%) cases. Also, 39% (95% CI: 34%-44%) of the new mothers did not breastfeed their newborns in the first hour after delivery. Thirty-three health workers attended the deliveries under this arm of the study. The number of deliveries attended by each health worker ranged from one to thirty-seven, with an average of 12 (S.D: 10) deliveries each and a median of eight during the observation period. At least two health workers attended about 61% of the 393 deliveries. Also, Junior Community Health Extension Workers (JCHEWs) attended the majority (36%) of the 393 deliveries (Table 1).

Table 1. Pregnant Women's Profile

For peer review only

	Quantitative arm % n=393	Qualitative arms % n=27
Age		
<15	0	3
15 - 24	53	63
25 - 34	36	30
35+	11	4
Gestational age in weeks		
Mean	41 (SD=12)	38(SD=0.6)
Mode	38	38
Median	38	38
Parity		
Nulliparous (first pregnancy above 28wks gestational age (GA))	20	33
Multiparous (more than first pregnancy above 28wk GA)	80	67
Initiated breastfeeding within the first hour after birth		
no	39	37
yes	61	63
Health worker who attended pregnant woman's labor and delivery		
nurse/midwife	4	0
junior community health extension worker	36	28
community health extension worker	18	15
environmental health assistant/technician/officer	28	19
hospital assistant	10	19
nutritionists/dieticians/students	4	19
Sex of health worker that attended pregnant woman's labor and delivery		
male	1	0
female	99	100

Under the qualitative arm, 27 pregnant women were delivered of their babies by 16 health workers. The pregnant women consented to be observed and interviewed. The 16 health workers also consented to be interviewed after the observation. Most (63%) of the pregnant women were between 15 and 24 years old, with a median age of 22 years. A quarter were below 20 years of age and a quarter above 30 years of age. None had a twin delivery. Also, 37% (95% CI: 19%-56%) of the mothers did not breastfeed the newborn within one hour of birth under this arm of the

For peer review only

study. We also interviewed all 27 mothers and 16 health workers one hour after childbirth. Of the 16 health care workers interviewed under the qualitative arm, most (63%) were hospital assistants. About a third (33%) were Community Health Extension Workers (CHEWS), 17% (2) were students, 6% (1) were nutritionist, and 6% (1) environmental health technician. Of the 27 deliveries under this arm of the study, in 44% of cases, the attending health care worker was assisted by another health worker (Table 1).

Knowledge of time to initiate breastfeeding

The qualitative arm shows that health workers know when mothers should breastfeed newborns. Ninety-two percent of them responded that breastfeeding should start between zero to sixty minutes (Table 2).

Table 2. Health Workers' Response to When Breastfeeding Should Start.

**Attending health care workers interviewed under qualitative arm**	**Frequency**	**Percent** *(n=16)*
Response to when breastfeeding should start		
Immediately after delivery	9	54
**10 - 30 minutes after delivery**	2	13
**30 - 60 minutes after delivery**	1	8
**0 - 60 minutes after delivery**	3	17
**0 - 24 hours after delivery**	1	8

They attributed their knowledge to on-the-job training sessions on newborn care with visiting clinical-mentors. They believe their knowledge is sustained via peer-to-peer discussions while on the job and during staff meetings. One Junior Community Health Extension Worker (JCHEW) said:

"breast feeding should commence immediately after birth …we have clinical mentors that visit and remind us of these things. We also have staff meetings where we remind ourselves of these practices" (birth attendant 1)

The word "immediately" is more used by lower cadre health workers to describe when breastfeeding should start. The CHEWs are more specific about when breastfeeding should start. They also link the time breastfeeding should start with its benefits. The quote below is a typical response from a Senior CHEW when she was asked when breastfeeding should start.

For peer review only

"It is very important because it helps the child to suck the yellowish nutrient in the breast milk. It boosts the child's immunity. It also helps the mother's uterus to shrink and close …helping to stop bleeding. It should start by 30min to 1hour after delivery" (birth attendant 2).

Barriers to early breastfeeding

Birth attendants' unwillingness or inability to accommodate mothers' safe traditional practices

The quantitative study shows that pregnant women denied safe traditional birth practices such as praying or reading religious texts during the second and third stages of labor are five times more likely not to breastfeed within the first hour (RR=4.5, 95% CI=1.2-17.1) compared to pregnant women allowed these practices (Table 3).

Table 3: Association Between Early Breastfeeding and Predictor Variables in the Study

Variable	Response	Women who didn't initiate breastfeeding within 1hr after birth		
		% n=154	*Relative risk 95% CI*	*p value*
Birth attendant had received training on newborn care	No	49	1.2 (0.9-1.5)	0.2
	Yes	51		
There were delays in providing care	No	90	0.7 (0.5-1.03)	0.2
	Yes	10		
Communication was easy and frequent between woman and birth attendant	No	2	0.7 (0.3-1.8)	0.5
	Yes	98		
Poor staff attitude	No	96	0.7 (0.4-1.1)	0.2
	Yes	4		
Woman denied some safe traditional childbirth practices*	No	99	4.5 (1.2-17.1)	0.003***
	Yes	1		
Birth attendant determined the birth position	No	41	1 (0.8-1.3)	>0.9
	Yes	59		
Woman was allowed to give birth in the position she preferred**	No	37	0.9 (0.7-1.2)	0.5
	Yes	63		
Woman encouraged to consume fluids/food at least once during labor	No	16	2.1 1.5-3.0	0.001
	Yes	84		
Mother and newborn kept in the same room after delivery (rooming-in)	No	2	2.6 2.3-2.9	0.059***
	Yes	98		
Mother had skin-to-skin contact with newborn in the	No	45	2.3	<0.001

For peer review only

first hour after birth	Yes	55	1.8-2.8	
Woman had blood loss greater than 500mls during labor and delivery	No	99	1.17<br>0.2-5.9	>0.9***
	Yes	1		
The labor room was clean and comfortable	No	18	0.98<br>(0.7-1.4)	0.9
	Yes	82		

**n=154, **n=150, ***Fishers* X^2

When mother's state of health after childbirth isn't an issue, a typical response given by some mothers for not breastfeeding in the first hour was the need to first wash the breast or have a bath to feel clean.

"he (the baby) has to exercise patience until we get home (before he is breastfed). I can't breastfeed him before I take my bath" (mother 1)

The quantitative study suggests that the mother's need to be clean may not be influenced by the cleanliness of the environment. Initiating breastfeeding is not related to the cleanliness and comfort provided by the delivery room. Mothers who had their babies when the labor room was clean and comfortable were just as likely not to initiate breastfeeding early as those who had their babies when the labor room was not (RR=0.98; 95% CI 0.7-1.4; p=0.9).

Poor management of mothers' post-delivery state of health

Post-delivery pains and fatigue are barriers to breastfeeding within the first hour after birth. Even when mothers show a good knowledge of when breastfeeding should start, some still express the need to regain strength and wellness first before they breastfeed the newborn.

"breastfeeding should commence immediately after birth… I did not commence it because I was feeling after pains. The health worker said she will bring the baby to suck. I told her to allow me to have some relief" ZA (mother 2)

The quantitative study shows that pregnant women not encouraged to consume fluids or food at least once during labor are twice as likely not to breastfeed within the first hour compared with those encouraged to do so (RR=2.1; 95% CI=1.5-3; p=0.001). There is no evidence from the study that blood loss greater than 500mls during labor and delivery influence early breastfeeding (RR=1.17; 95% CI=0.2-5.9; p=1).

Human resource shortages

For peer review only

The qualitative study shows that shortage of health workers in PHCs introduces a delay in carrying out newborn care activities. Sometimes, health workers have to attend to other ill patients when there is no one else to assist. When we asked some health workers why a mother under their care did not breastfeed early, a typical response given was:

““You see, if you have someone that will assist you, you will assign the person to carry the baby to the mother and initiate the breastfeeding, or weigh the child, or apply chlorhexidine to the baby’s cord or any other thing needed while you continue with the remaining work and management of others …but most of the time you are on duty alone. It is because we have shortage of manpower here in this facility” (birth attendant 3)

The human resource shortage also affects rooming-in in the PHCs. Placing the mother and newborn in the same room after delivery is rooming-in[52]. Sometimes, the health worker has to clean and make this room ready for the mother and newborn. When there is a shortage of staff, this delays the transfer of the mother and newborn to the rooming-in room, and affects early breastfeeding.

“I wanted to transfer the mother and baby to the postnatal ward… before she commences breastfeeding (of the newborn). The (lying-in) room is not set (for use yet). I have to clean and make the room so she is comfortable to commence breastfeeding” (birth attendant 4)

Ineffective rooming-in practices

When rooming-in happens, the quantitative study reveals that mothers who do not have skin-to skin contact with their newborns in the first hour after birth are twice as likely not to breastfeed early, compared to mothers who did (RR=2.3, 95% CI=1.8-2.8; p<0.001). Just keeping the mother and newborn in the same room (rooming-in) without skin-to-skin contact has no influence on early breastfeeding (RR=2.6; 95% CI=2.3-2.9; p=0.059).

Lack of privacy and proper visiting-hour policy in the PHCs

The PHCs have open rooming-in rooms that doesn’t guarantee privacy. There are also no defined visiting hours in the PHCs. The qualitative arm of the study shows that male and female relatives visit the new mother in the hospital after childbirth. During the visit, the relatives pray for the newborn baby and congratulate the mother. Some relatives sit around after prayers for long. When relatives come visiting, the mothers have to dress up to receive them and do not breastfeed

For peer review only

during this time. The birth attendants also delay supporting the mother to breastfeed. One birth attendant suggested that addressing the issue puts them in bad light in the community.

"you know, the people in the community have a unique character or attitude. The moment you try to talk to them about this kind of issue they feel you are molesting them or depriving them of coming close to their relatives. They do not know you are trying to ensure their relative (mother and baby) gets what is beneficial to them" (birth attendant 5).

Facilitators of early breastfeeding

Health education during ANC and post-delivery period

From the qualitative study, we find that Antenatal Clinics (ANC) helps pregnant women to learn about breastfeeding newborns within one hour of birth. The knowledge they gain during these clinics influences their behavior after childbirth. Most mothers who practiced early breastfeeding said what they learned from ANC influenced their decision to do so. The typical response they gave is:

"I used to give my children water (after childbirth). I did not know the importance of breastfeeding early. I used to think breast feeding could start at any time of the day (of birth). I started breastfeeding early because the health workers tell us (of the importance of starting breastfeeding immediately after birth) during ANC" (mother 3)

Encouraging and supporting mothers to start breastfeeding after childbirth

From the qualitative study, we find that when the birth attendants encourage some reluctant mothers to breastfeed their newborns within one hour of childbirth; they do.

"I was told (by the health worker) to give (the baby breast to suck early enough). Normally, I won't" (mother 4)

The encouragement process takes the form of a negotiation between the birth attendant and the mother. We find that the information passed by the birth attendants to the mothers around the benefits of early breastfeeding, facilitate early breastfeeding in the PHCs.

"I commenced breastfeeding early because of what the health worker said …she explained how it is important to the health of my baby. That's why I commenced it" (mother 5)

For peer review only

Sometimes, showing the mothers how to place and breastfeed the newborns also facilitates early breastfeeding after childbirth in these health facilities.

"I gently encourage them and tell them to give (breast milk) early… I put the baby on her laps and remove the breast, and demonstrate to her how to breastfeed" (birth attendant 6)

DISCUSSION

In our study setting, we find that close to four out of every 10 newborns do not get breastfed within the first hour of birth. This doubles their risk of dying in the first 28 days of their lives[10,15]. Our estimate is four percentage points lower than what researcher observed in PHCs in Asia[31]. It is also higher than estimates amongst mothers who had spontaneous vaginal deliveries (SVD) in some secondary health facilities; and lower than estimates amongst mothers who had SVD in some tertiary health facilities, even in Nigeria[31,53,54]. The mixed results emphasize the influence of context on the early breastfeeding of newborns in different health facilities. It underscores the need for unique interventions to address the problem.

The northeast region of Nigeria has a shortage of skilled health workers. The Boko-Haram insurgency has made this worse[55–57]. It is also worsened by staff absenteeism in PHCs in the region. Only about 35% of employed staff in PHCs in Gombe are likely to be at work on any given day, for example[22]. Studies suggest that a shortage of human resource and a dominant population of unskilled health care workers affects the quality of newborn care in health facilities[17,58–60]. This is not overall consistent with our study findings. We find that unskilled health workers are dominant in our study setting. Also, we find that human resource shortage is a barrier to early initiation of breastfeeding in the PHCs. We did not find that the skills of the birth attendants influenced the early breastfeeding of newborns. This may be because clinical mentors have trained the birth attendants in our study on newborn care. Educational interventions around support for the breastfeeding of newborns has been found to improve health workers' knowledge, attitude, and compliance with the practice[61,62].

In our study, we find that the mothers denied safe traditional birth practices like praying, reciting religious texts, or reading religious books during deliveries were five times more likely not to breastfeed the newborns than the mothers not denied. This supports the findings from other settings that shows that the unwillingness of birth attendants to accommodate safe traditional birth practices affects mothers' adoption of supportive care[59,63,64]. This may be because mothers

For peer review only

perceive denial of such traditional practices as mistreatment or abuse[59,65]. A study in Norway finds that the recent abuse of women by “both known and unknown” perpetrators affects their breastfeeding behavior[66]. Although in our study, we found no evidence to support this. Instead we find that birth attendants’ attitude, rapport skills, and negligence during the delivery period doesn’t affect early breastfeeding practice amongst mothers. The discrepancy in findings may be because the women in our study setting perceive or tolerate abuse differently than women in other settings[67].

Washing of the breast with water after childbirth is a hygiene related practiced in parts of the world[68]. In our study, mothers express a strong need to wash the breast and/or have a bath before breastfeeding newborns. This makes the “need for a bath after childbirth” a major theme for why mothers delay breastfeeding newborns in our study setting. Some health workers also agree that mothers should wash their breasts first before breastfeeding newborns after childbirth[18]. Their inability to help the mothers to wash their breasts or have a bath before breastfeeding may be because there is no running water in the health facility. Only 38% of priority PHCs in Gombe have running water[22]. The need to wash the breast may be deeper than the need for hygiene, nonetheless. Washing of the breast or having a bath after childbirth before breastfeeding newborns has traditional and/or religious undertones in other parts of the world[60,69].

The other barriers that influence early breastfeeding of newborn in PHCs that our study finds are post-delivery pains and fatigue within the first hour after birth, delay in skin to skin contact during rooming-in, and poor visiting hour policy in the PHC. These findings reinforces what other studies have reported[5,21,24,33,41,54,60]. The post-delivery fatigue may be because the mother is famished. This may also be due to anemia, infections, thyroid disorders, mood disorders and cardiomyopathy[70] which our study does not assess. The quantitative arm of our study shows that mothers not encouraged to take fluids or eat during the second and third stages of labor are more likely not to breastfeed early after childbirth. There is no evidence from our study that the fatigue may be due to blood loss. We also find that rooming-in is not enough and that skin to skin contact must be deliberate. Mothers that did not have skin to skin contact with their newborns while in the rooming-in room were more likely not to breastfeed than those who did in our study. Our study reemphasizes that the lack of restrictions on relatives visiting the mothers in the post-delivery period impedes breastfeeding[71]. This could be because the mothers don’t feel comfortable breastfeeding in front of male relatives or visitors.

For peer review only

Helping mothers to breastfeed within an hour of birth is an international recommendation practiced by health care providers in most of the world[32,72]. We find that birth attendants in our study setting practice this and the strategy facilitates the early breastfeeding of newborns in public PHCs. This may be because there is an awareness of the recommendation[73]. Our study also reinforces findings from previous studies on the benefits of health education on the timely breastfeeding of newborns[74,75]. In our study, we find that health education received by mothers during ANC and the post-delivery period improves the practice of early initiation of breastfeeding in public PHCs, in our study setting

CONCLUSION

Only about 60% of babies born in public PHCs in Northeast Nigeria get breastfed in the first hour of birth. This means the rest miss important nutrition that saves lives. The stakeholders in the region must increase their focus on improving the breastfeeding practices in public PHCs. This will improve the survival of newborns and impact of their investments. Instituting policies that protect mothers' privacy; and finding innovative ways to accommodate and promote safe traditional practices in the intrapartum and postpartum period in PHCs will improve the early breastfeeding of newborns in these PHCs. Birth attendants in PHCs must also be trained on effective rooming-in to further improve early breastfeeding of newborns in these public PHCs, however.

LIST OF ABBREVIATIONS

PHC (Primary Health Care hospital/facility), ANC (Antenatal Care) VHW (Village Health Workers), CHEWs (Community Health Extension Workers).

DECLARATIONS

Ethics approval and consent to participate

The Gombe State Ministry of Health Ethics Committee granted ethical approval for the qualitative arm of the study (reference no: MOH/ADM/658/VOL.II/104). Mothers and birth attendants also gave their consent to take part in the study before the interviews. The IDEAS team at the London School of Hygiene and Tropical Medicine (LSHTM) got ethical approval for the quantitative arm of the study from LSHTM (reference 6088).

Funding

For peer review only

The study is funded as part of the evaluation of SFH's MNCH2 project being funded by the Bill and Melinda Gates Foundation and implemented in Gombe State. The views and opinions expressed in the paper are those of the authors and do not reflect those of SFH and/or GSPHCDA. The intervention is funded through donor grants received by SFH. The donor did not play a role in the design of this evaluation.

Data Availability

Data for the quantitative arm of the study is in a secured database at the London School of Hygiene and Tropical Medicine. Write to Nasir Umar through nasir.umar@lshtm.ac.uk to place a reasonable request for the anonymized version of it. Also, write to Shobo Olukolade, through shoboolukolade@gmail.com to ask for the anonymized transcripts of the qualitative data.

Competing interest

OGS and PL are consultants working for Society for Family Health (SFH), OI and JA are full-time staff of SFH. GA is the Former Executive Secretary of Gombe State Primary Health Care Development Agency. SFH's program in Northeast Nigeria seeks to improve MNCH outcomes in the general population.

Author Contribution

OGS conceived of the study and developed the original draft of the manuscript. OGS analyzed the quantitative data. OGS and PL analyzed the qualitative data. NU, GA, JA, and OI reviewed, edited, and made significant contributions to the development of the final manuscript. All authors read and approved the final manuscript.

Acknowledgement

For peer review only

We thank all the staff of SFH MNCH2 project in Gombe, particularly Mrs. Magdalene Okolo for their contributions to the study. We also thank the field staff who conducted the observations and interviews. We also thank our partners, the members of the IDEAS team from the London School of Hygiene and Tropical Medicine, for leadership in designing and implementing the quantitative arm of the study.

REFERENCES

1. World Health Organization. *Reaching the every newborn national 2020 milestones: country progress, plans and moving forward*. http://apps.who.int/iris/bitstream/handle/10665/255719/9789241512619-eng.pdf?sequence=1 (2017).
2. Edmond, K. M. *et al.* Delayed Breastfeeding Initiation Increases Risk of Neonatal Mortality. *Pediatrics* **117**, e380–e386 (2006).
3. Mullany, L. C. *et al.* Breast-Feeding Patterns, Time to Initiation, and Mortality Risk among Newborns in Southern Nepal. *J Nutr* **138**, 599–603 (2008).
4. World Health Organization. *Every newborn: an action plan to end preventable deaths*. (World Health Organization, 2014).
5. WHO | Early initiation of breastfeeding to promote exclusive breastfeeding. *WHO* http://www.who.int/elena/titles/early_breastfeeding/en/.
6. WHO | Newborn care at birth. *WHO* http://www.who.int/maternal_child_adolescent/newborns/care_at_birth/en/.
7. Debes, A. K., Kohli, A., Walker, N., Edmond, K. & Mullany, L. C. Time to initiation of breastfeeding and neonatal mortality and morbidity: a systematic review. *BMC Public Health* **13**, S19 (2013).

For peer review only

8. UNICEF & WHO. *Capture the moment: early initiation of breastfeeding : the best start for every newborn*. (2018).

9. Friedrich, M. J. Early Initiation of Breastfeeding. *JAMA* **320**, 1097–1097 (2018).

10. al, K. J., et. Timing of breastfeeding initiation and exclusivity of breastfeeding during the first month of life: effects on neonatal mortality and morbidity--a ... - PubMed - NCBI. https://www.ncbi.nlm.nih.gov/pubmed/24894730.

11. Federal Ministry of Health. Nigeria Every Newborn Action Plan: A plan to end preventable newborn deaths in Nigeria. (2016).

12. Anyamele, O., Akanegbu, B., Assad, J.-C. & Ukawuilulu, J. Differentials in Infant and Child Mortality in Nigeria: Evidence from Pooled 2003 and 2008 DHS Data. *Advances in Management and Applied Economics* **7**, 73–96 (2017).

13. Morakinyo, O. M. & Fagbamigbe, A. F. Neonatal, infant and under-five mortalities in Nigeria: An examination of trends and drivers (2003-2013). *PLOS ONE* **12**, e0182990 (2017).

14. Tura, G., Fantahun, M. & Worku, A. The effect of health facility delivery on neonatal mortality: systematic review and meta-analysis. *BMC Pregnancy Childbirth* **13**, 18 (2013).

15. Khan, J., Vesel, L., Bahl, R. & Martines, J. C. Timing of Breastfeeding Initiation and Exclusivity of Breastfeeding During the First Month of Life: Effects on Neonatal Mortality and Morbidity—A Systematic Review and Meta-analysis. *Matern Child Health J* **19**, 468–479 (2015).

16. Takahashi, K. *et al.* Prevalence of early initiation of breastfeeding and determinants of delayed initiation of breastfeeding: secondary analysis of the WHO Global Survey. *Scientific Reports* **7**, 44868 (2017).

For peer review only - http://bmjopen.bmj.com/site/about/guidelines.xhtml

17. Haile, Z. T. *et al.* The Moderating Effect of Urban Versus Rural Residence on the Relationship Between Type of Birth Attendant and Early Initiation of Breastfeeding in Ghana. *J Hum Lact* **34**, 810–820 (2018).

18. Bee, M., Shiroor, A. & Hill, Z. Neonatal care practices in sub-Saharan Africa: a systematic review of quantitative and qualitative data. *Journal of Health, Population and Nutrition* **37**, 9 (2018).

19. Sobel, H. L., Silvestre, M. A. A., Iii, J. B. V. M., Oliveros, Y. E. & Nyunt-U, S. Immediate newborn care practices delay thermoregulation and breastfeeding initiation. *Acta Paediatrica* **100**, 1127–1133 (2011).

20. Adewuyi, E. O., Zhao, Y., Khanal, V., Auta, A. & Bulndi, L. B. Rural-urban differences on the rates and factors associated with early initiation of breastfeeding in Nigeria: further analysis of the Nigeria demographic and health survey, 2013. *International Breastfeeding Journal* **12**, 51 (2017).

21. Berde, A. S. & Yalcin, S. S. Determinants of early initiation of breastfeeding in Nigeria: a population-based study using the 2013 demograhic and health survey data. *BMC Pregnancy and Childbirth* **16**, 32 (2016).

22. Marchant, T. *Change in maternal and newborn health care interactions between families and frontline workers. Report from Gombe State, Nigeria, 2012-2015*. https://ideas.lshtm.ac.uk/wp-content/uploads/2017/08/Marchant_IDEAS_Gombe_State_Follow-upSurvey_2016.pdf (2016).

23. Thulier, D. & Mercer, J. Variables Associated With Breastfeeding Duration. *Journal of Obstetric, Gynecologic & Neonatal Nursing* **38**, 259–268 (2009).

For peer review only

24. Ahmed, A. E. & Salih, O. A. Determinants of the early initiation of breastfeeding in the Kingdom of Saudi Arabia. *International Breastfeeding Journal* **14**, 13 (2019).

25. Liben, M. L. & Yesuf, E. M. Determinants of early initiation of breastfeeding in Amibara district, Northeastern Ethiopia: a community based cross-sectional study. *International Breastfeeding Journal* **11**, 7 (2016).

26. Delivery care. *UNICEF DATA* https://data.unicef.org/topic/maternal-health/delivery-care/.

27. Bohren, M. A. *et al.* Facilitators and barriers to facility-based delivery in low- and middle-income countries: a qualitative evidence synthesis. *Reproductive Health* **11**, 71 (2014).

28. Campbell, O. M. & Graham, W. J. Strategies for reducing maternal mortality: getting on with what works. *The Lancet* **368**, 1284–1299 (2006).

29. WHO | Early initiation of breastfeeding. *WHO* http://www.who.int/elena/titles/commentary/early_breastfeeding/en/.

30. Forster, D. A. & McLachlan, H. L. Breastfeeding Initiation and Birth Setting Practices: A Review of the Literature. *Journal of Midwifery & Women's Health* **52**, 273–280 (2007).

31. Karim, F. *et al.* Initiation of breastfeeding within one hour of birth and its determinants among normal vaginal deliveries at primary and secondary health facilities in Bangladesh: A case-observation study. *PLOS ONE* **13**, e0202508 (2018).

32. WHO | Protecting, promoting and supporting breastfeeding in facilities providing maternity and newborn services. *WHO* http://www.who.int/elena/titles/full_recommendations/breastfeeding-support/en/.

33. Sharma, I. K. & Byrne, A. Early initiation of breastfeeding: a systematic literature review of factors and barriers in South Asia. *International Breastfeeding Journal* **11**, 17 (2016).

For peer review only

34. DiGirolamo, A. M., Grummer-Strawn, L. M. & Fein, S. Maternity Care Practices: Implications for Breastfeeding. *Birth* **28**, 94–100 (2001).

35. Fedreal Ministry of Health. Nigeria Investment case for reproductive, maternal, newborn, child, adolescent health and nutrition: 2017 - 2030. (2017).

36. The Maternal and Neonatal Health Project in North East Nigeria 2012 – 2016 | Society for Family Health, Nigeria. http://www.sfhnigeria.org/the-maternal-and-neonatal-health-project-in-north-east-nigeria-2012-2016/.

37. State Accountability and Quality Improvement Project. *Pact* https://www.pactworld.org/country/nigeria/project.

38. Strengthening the healthcare system in north-east Nigeria - a priority for WHO | WHO | Regional Office for Africa. https://www.afro.who.int/news/strengthening-healthcare-system-north-east-nigeria-priority-who.

39. Makinde, O. A., Sule, A., Ayankogbe, O. & Boone, D. Distribution of health facilities in Nigeria: Implications and options for Universal Health Coverage. *The International Journal of Health Planning and Management* https://onlinelibrary.wiley.com/doi/abs/10.1002/hpm.2603 (2018) doi:10.1002/hpm.2603.

40. Nigeria Health Facility Registry. https://hfr.health.gov.ng/facilities/search?_token=r2vSeMamYxcYqkwMtdRF67CgGTWkRNkpYS6QoUQV&facility_type_id=1&state_id=115&lga_id=&ward_id=0&facility_level_id=2&ownership_id=1&operational_status_id=0&registration_status_id=0&license_status_id=0&geo_codes=0&service_type=0&service_category_id=0&facility_name=.

41. Bbaale, E. Determinants of Early Initiation, Exclusiveness, and Duration of Breastfeeding in Uganda. *J Health Popul Nutr* **32**, 249–260 (2014).

For peer review only

42. Li, R., Scanlon, K. S. & Serdula, M. K. The Validity and Reliability of Maternal Recall of Breastfeeding Practice. *Nutr Rev* **63**, 103–110 (2005).

43. Kawulich, B. B. Participant Observation as a Data Collection Method. *Forum Qualitative Sozialforschung / Forum: Qualitative Social Research* **6**, (2005).

44. Altmann, J. Observational Study of Behavior: Sampling Methods. *Behaviour* **49**, 227–267 (1974).

45. Schoonenboom, J. & Johnson, R. B. How to Construct a Mixed Methods Research Design. *Kolner Z Soz Sozpsychol* **69**, 107–131 (2017).

46. Denzin, N. K. & Lincoln, Y. S. *The SAGE Handbook of Qualitative Research*. (SAGE, 2011).

47. Gombe State Government. Gombe State Nigeria: History. *Gombe State* http://gombestate.gov.ng/history-2/.

48. Federal Ministry of Health. Federal Republic of Nigeria Draft National Human Resources for Health Strategic Plan 2008 - 2012. (2012).

49. Alenoghena, I. O., Isah, E. C. & Isara, A. R. Availability and Type of Human Resource for Health in Public Primary Health Care Facilities in Selected Communities, Edo State. *Journal of Community Medicine and Primary Health Care* **28**, 11–18 (2016).

50. Marchant, D. T. Maternal and newborn health care. Baseline findings from Gombe State, Nigeria. https://ideas.lshtm.ac.uk/report/mnh-care-baseline-gombe-state/ (2013) doi:10.17037/PUBS.01126638.

51. Patton, M. Designing Qualitative Studies. in *Qualitative evaluation and research methods* 169–186 (SAGE, 1990).

52. Jaafar, S. H., Ho, J. J. & Lee, K. S. Rooming-in for new mother and infant versus separate care for increasing the duration of breastfeeding. *Cochrane Database Syst Rev* CD006641–CD006641 (2016) doi:10.1002/14651858.CD006641.pub3.

For peer review only

53. Örün, E. *et al.* Factors associated with breastfeeding initiation time in a Baby-Friendly Hospital. *The Turkish Journal of Pediatrics* **52**, 10–16 (2010).

54. Awi, D. D. & Alikor, E. a. D. Barriers to timely initiation of breastfeeding among mothers of healthy full-term babies who deliver at the University of Port Harcourt Teaching Hospital. *Nigerian Journal of Clinical Practice* **9**, 57-64–64 (2006).

55. Federal Ministry of Health. National Human Resource for Health Strategic Plan: 2008 - 2012. (2008).

56. Premium Times Nigeria. Gombe govt. waives retirement for 100 nurses, midwives due to manpower shortage. https://www.premiumtimesng.com/news/more-news/230165-gombe-govt-waives-retirement-100-nurses-midwives-due-manpower-shortage.html (2017).

57. Omoluabi, E. *Needs Assessment of the Nigerian Health Sector*. https://nigeria.iom.int/sites/default/files/newsletter/ANNEX%20XXIV%20Needs%20Assessment%20of%20the%20Nigeria%20health%20Sector.pdf (2014).

58. Aluko, J. O., Anthea, R. & Marie Modeste, R. R. Manpower capacity and reasons for staff shortage in primary health care maternity centres in Nigeria: a mixed-methods study. *BMC Health Services Research* **19**, 10 (2019).

59. Bohren, M. A. *et al.* The Mistreatment of Women during Childbirth in Health Facilities Globally: A Mixed-Methods Systematic Review. *PLoS Med* **12**, e1001847 (2015).

60. Majra, J. P. & Silan, V. K. Barriers to Early Initiation and Continuation of Breastfeeding in a Tertiary care Institute of Haryana: A Qualitative Study in Nursing Care Providers. *J Clin Diagn Res* **10**, LC16–LC20 (2016).

61. Balogun, O. O. *et al.* Health Facility Staff Training for Improving Breastfeeding Outcome: A Systematic Review for Step 2 of the Baby-Friendly Hospital Initiative. *Breastfeeding Medicine* **12**, 537–546 (2017).

For peer review only

62. Okolo, S. N. & Ogbonna, C. Knowledge, attitude and practice of health workers in Keffi local government hospitals regarding Baby-Friendly Hospital Initiative (BFHI) practices. *Eur J Clin Nutr* **56**, 438–441 (2002).

63. Mannava, P., Durrant, K., Fisher, J., Chersich, M. & Luchters, S. Attitudes and behaviours of maternal health care providers in interactions with clients: a systematic review. *Globalization and Health* **11**, 36 (2015).

64. Chatters, L. M. Religion and Health: Public Health Research and Practice. *Annual Review of Public Health* **21**, 335–367 (2000).

65. Beck, C. T. A Secondary Analysis of Mistreatment of Women During Childbirth in Health Care Facilities. *Journal of Obstetric, Gynecologic & Neonatal Nursing* **47**, 94–104 (2018).

66. Sørbø, M. F., Lukasse, M., Brantsæter, A.-L. & Grimstad, H. Past and recent abuse is associated with early cessation of breast feeding: results from a large prospective cohort in Norway. *BMJ Open* **5**, e009240 (2015).

67. Maya, E. T. *et al.* Women's perspectives of mistreatment during childbirth at health facilities in Ghana: findings from a qualitative study. *Reproductive Health Matters* **26**, 70–87 (2018).

68. Khan, M. E. Shaping Demand and Practices to Improve Family Health Outcomes: Findings from a Quantitative Survey, Volume 3: Bihar. 221.

69. Tawiah-Agyemang, C., Kirkwood, B. R., Edmond, K., Bazzano, A. & Hill, Z. Early initiation of breast-feeding in Ghana: barriers and facilitators. *Journal of Perinatology* **28**, S46–S52 (2008).

70. Atkinson, L. S. & Baxley, E. G. Postpartum fatigue. *Am Fam Physician* **50**, 113–118 (1994).

For peer review only

71. Saxena, M., Srivastava, A., Dwivedi, P. & Bhattacharyya, S. Is quality of care during childbirth consistent from admission to discharge? A qualitative study of delivery care in Uttar Pradesh, India. *PLOS ONE* **13**, e0204607 (2018).

72. UNICEF & World Health Organization. Country Experiences with the Baby-Friendly Hospital Initiatiive. Compendium of Case Studies from Around the World. (2017).

73. Kakrani, V. A., Rathod (Waghela), H. K., Mammulwar, M. S. & Bhawalkar, J. S. Awareness about "Ten Steps for Successful Breastfeeding" among Medical and Nursing Students. *Int J Prev Med* **6**, (2015).

74. Guise, J.-M. *et al.* The Effectiveness of Primary Care-Based Interventions to Promote Breastfeeding: Systematic Evidence Review and Meta-Analysis for the US Preventive Services Task Force. *Ann Fam Med* **1**, 70–78 (2003).

75. de Oliveira, M. I. C., Camacho, L. A. B. & Tedstone, A. E. Extending Breastfeeding Duration Through Primary Care: A Systematic Review of Prenatal and Postnatal Interventions. *J Hum Lact* **17**, 326–343 (2001).

For peer review only

Factors influencing the early initiation of breastfeeding in public primary health care facilities in Northeast Nigeria: A mixed methods study

Journal:	*BMJ Open*
Manuscript ID	bmjopen-2019-032835.R3
Article Type:	Research
Date Submitted by the Author:	10-Mar-2020
Complete List of Authors:	Shobo, Olukolade; Society for Family Health , Monitoring and Evaluation Department<br>Umar, Nasir; London School of Hygiene and Tropical Medicine Faculty of Infectious and Tropical Diseases, Disease Control<br>Gana, Ahmed; Gombe State Primary Health Care Development Agency, Office of the Executive Secretary<br>Longtoe, Peter; Society for Family Health , Monitoring and Evaluation Department<br>Idogho, Omokhudu; Society for Family Health , Office of the Managing Director<br>Anyanti, Jennifer; Society for Family Health , Office of the Deputy Managing Director
**Primary Subject Heading**:	Health services research
Secondary Subject Heading:	Nutrition and metabolism, Obstetrics and gynaecology
Keywords:	Health policy < HEALTH SERVICES ADMINISTRATION & MANAGEMENT, Quality in health care < HEALTH SERVICES ADMINISTRATION & MANAGEMENT, PRIMARY CARE, NEONATOLOGY

SCHOLARONE™
Manuscripts

I, the Submitting Author has the right to grant and does grant on behalf of all authors of the Work (as defined in the below author licence), an exclusive licence and/or a non-exclusive licence for contributions from authors who are: i) UK Crown employees; ii) where BMJ has agreed a CC-BY licence shall apply, and/or iii) in accordance with the terms applicable for US Federal Government officers or employees acting as part of their official duties; on a worldwide, perpetual, irrevocable, royalty-free basis to BMJ Publishing Group Ltd ("BMJ") its licensees and where the relevant Journal is co-owned by BMJ to the co-owners of the Journal, to publish the Work in this journal and any other BMJ products and to exploit all rights, as set out in our licence.

The Submitting Author accepts and understands that any supply made under these terms is made by BMJ to the Submitting Author unless you are acting as an employee on behalf of your employer or a postgraduate student of an affiliated institution which is paying any applicable article publishing charge ("APC") for Open Access articles. Where the Submitting Author wishes to make the Work available on an Open Access basis (and intends to pay the relevant APC), the terms of reuse of such Open Access shall be governed by a Creative Commons licence – details of these licences and which Creative Commons licence will apply to this Work are set out in our licence referred to above.

Other than as permitted in any relevant BMJ Author's Self Archiving Policies, I confirm this Work has not been accepted for publication elsewhere, is not being considered for publication elsewhere and does not duplicate material already published. I confirm all authors consent to publication of this Work and authorise the granting of this licence.

For peer review only

Factors influencing the early initiation of breastfeeding in public primary health care facilities in Northeast Nigeria: A mixed methods study

Corresponding Author:

1. Olukolade George Shobo (Society for Family Health, 8 Port Harcourt Crescent, Area 11, Garki, Abuja. Email: shoboolukolade@gmail.com)

Coauthors:

2. Nasir Umar (London School of Hygiene and Tropical Medicine, London. Email: nasir.umar@lshtm.ac.uk)
3. Gana Ahmed (Gombe State Primary Health Care Development Agency. Gombe State. Email: ahmedgana567@yahoo.com)
4. Peter Longtoe (Society for Family Health, 8 Port Harcourt Crescent, Area 11, Garki, Abuja. Email: plongtoe.com)
5. Omokhudu Idogho (Society for Family Health, 8 Port Harcourt Crescent, Area 11, Garki, Abuja. Email: oidogho@sfhnigeria.org)
6. Jennifer Anyanti (Society for Family Health, 8 Port Harcourt Crescent, Area 11, Garki, Abuja. Email: janyanti@sfhnigeria.org)

ABSTRACT

Introduction

The early initiation of breastfeeding is a high-impact intervention that gives newborns a better chance of survival. We assess the barriers and facilitators influencing the practice of early breastfeeding of newborns in public primary healthcare facilities (PHCs) in Northeast Nigeria, to influence the planning of programs targeted at improving newborn care in the region.

Method

We used an explanatory mixed methods approach. We conducted case-observation of childbirths and newborn care for the quantitative arm, and interviewed mothers and birth attendants one hour after childbirth for the qualitative arm. The analysis for the quantitative arm was done with SPSS version 23. For the qualitative arm, we transcribed the audio files, coded the texts, and categorized them using thematic analysis.

Result

We observed 393 and 27 mothers for the quantitative and qualitative arms of the study respectively. The quantitative arm shows that 39% of mothers did not breastfeed their newborns within one hour of birth. The qualitative arm shows that 37% of mothers did not breastfeed within

For peer review only

one hour of birth. Themes that describe the barriers to early breastfeeding in public PHCs are: birth attendants' unwillingness or inability to accommodate mothers' safe traditional practices, ineffective rooming-in practices, staff shortages, lack of privacy in the lying-in ward, and poor implementation of visiting-hour policy in public PHCs. The pregnant women denied safe traditional birth practices like chanting, praying, or reading religious books during delivery are five times more likely not to breastfeed newborns within the first hour of birth (RR=4.5, 95% CI=1.2-17.1) compared to pregnant women allowed these practices.

Conclusion

Stakeholders must increase their focus on improving the breastfeeding practices in the public PHCs. Instituting policies that protect mothers' privacy; and finding innovative ways to accommodate and promote safe traditional practices in the intrapartum and postpartum period in PHCs will improve the early breastfeeding of newborns in these PHCs.

STRENGHTS AND LIMITATIONS OF THE STUDY

- We used a mixed methods study design
- Also, we used purposive sampling technique to select the public PHC, focusing on information-rich cases.
- Data collection included direct observation of mothers' newborn breastfeeding behavior in the first hour after childbirth
- We observed a high number of childbirths
- The sampling approach may limit the generalizability of the findings to places not similar to our study setting.

INTRODUCTION

Every year, about 77 million (50%) newborns do not get breastfed in the first hour of birth globally[1]. This leaves them vulnerable to diseases and death[1–3]. Newborn deaths continue to account for close to half of all under-5 mortalities across the world[4]. The early initiation of breastfeeding which means breastfeeding a newborn within one hour of birth[5], is a high-impact intervention[4,6,7] that gives newborns a better chance of survival[8]. It also provides them long-term health benefits[9,10]. The Northeast region of Nigeria has one of the highest newborn mortalities in

For peer review only

the world[11–13]. The early initiation of breastfeeding can reduce the risk of these newborn deaths by about a third[14,15].

The practice of early breastfeeding of newborns differs between and within countries[16,17]. For instance, it ranges between 17% to 95% for countries in sub-Saharan Africa [18,19]. In Nigeria, only about 35% of newborns get breastfed within the first hour after birth [20,21]. In the Northeast region of the country, only about 40% of mothers commence the breastfeeding of newborns in the first hour after childbirth[21]. In Gombe State, the estimate is 49%[22]. In the rural areas of the country, mothers are more likely not to practice it at all[20]. The mothers' age, level of education, and socioeconomic status are factors that influence the pattern of early initiation of breastfeeding in the general population. Others are maternal and newborn health problems, childbirth method, family support, availability of supplements, and maternal preference[21,23–25].

To improve early breastfeeding and avert newborn deaths, one approach is for more births to occur in health facilities[14,26–28]. Having a birth in a health facility improves the chance that a newborn will breastfeed early[21]. Evidence however suggests that poor birthing practices occur in health facilities which can disrupt the early start of breastfeeding[17,29–31]. While international recommendations for improving breastfeeding practices in health facilities exist[32], the problems faced by health facilities around the practice are context specific[33,34], requiring unique responses. Researches focusing on exploring and understanding these specific birthing practices in health facilities are now emerging[18,29].

The Northeast region of Nigeria is witnessing an increase in health investments by governments and non-governmental organizations[35–38]; aimed at reducing maternal and newborn deaths through improved access to quality childbirth and newborn care services in primary health care hospitals (PHCs)[35,36]. In Gombe State in Northeast Nigeria for instance, the government is implementing a Village Health Worker program that improves access to quality obstetric and newborn care services in public PHCs, through community-based demand generation activities. Primary health care facilities are better positioned to deliver high-impact newborn interventions in Nigeria. They make up 88% of health facilities in the country. In the Northeast, there are 5,086 public PHCs, and they make up 87% of the health facilities in the region[39,40]. Understanding the factors that influence the quality and uptake of life-saving newborn care services in these public PHCs is important for improving the effectiveness of the health investments in the region.

In this study, we assess the barriers and facilitators influencing early breastfeeding of newborns in public PHCs in Northeast Nigeria. Most studies assessing breastfeeding practice in health facilities have used secondary data from demographic health surveys[21,24,25,41]. While maternal recall is valid and reliable in establishing the period of initiation of breastfeeding[42], the DHS findings do not provide context concerning supply side factors that influenced the maternal behavior. A recent quantitative study in Bangladesh shows that about 43% of mothers in PHCs in the country do not breastfeed their newborns within one hour of birth. It did not assess the contextual implementation issues that influence the practice[31]. The literature around the contextual issues affecting the practice in PHCs in Africa is also lacking. Our approach uses a broader study design that helps to develop a deeper understanding of early breastfeeding practices in public PHCs in Northeast Nigeria[43,44]. We believe our findings will contribute to the discussions about health investments and strategies for improving newborn care in the region, and settings similar to it.

METHOD

Study Design

We used an explanatory mixed methods approach for this study. Mixed methods study designs deepen how we understand a research phenomenom[45,46]. In the explanatory mixed method type, a first phase quantitative data collection and analysis is followed by the collection of qualitative data, to explain the quantitative result[45]. The mixed-method approach helps us better understand the factors that influence the early initiation of breastfeeding in public PHCs[45]. We conducted the quantitative arm over four weeks in December 2017, and the qualitative arm over one week in November 2018. Budget constraints delayed implementing the qualitative arm. We assume that the time difference between the study arms is not sufficient to change the practice around breastfeeding newborns in the study setting.

Study Setting

We conducted the study in Gombe State, in the center of Northeast Nigeria on latitude 9” 30’ and 12” 30’N, Longitude 8” 5’and 11” 45’E. It borders Borno, Yobe, Adamawa, Taraba, and Bauchi State. It has 11 Local Government Areas (LGAs) and 114 political wards[47]. There are 603 health facilities across the 11 LGAs in the State, 530 of which are public PHCs[40]. Of the 530 public PHCs in the State, the government has designated 114 as priority PHCs. These 114 are Ward Health

For peer review only

Centers and provide basic emergency obstetric and newborn care services. Nurses, community health workers, community health extension workers (CHEWs), junior CHEWs, and environmental health officers are the main staff of PHCs in Nigeria[48]. Community Health Officer, a Public Health Nurse, three CHEWs, four Nurse/Midwives and one medical Assistant are the main staff of a Ward Health Center[49].

Fifty percent of these priority PHCs have staff trained in providing basic emergency obstetric and newborn care services. About 36% of them have labor rooms and lying-in wards. None has a medical doctor, 4% have at least one nurse, and 19% have at least one midwife. Each PHC has an average of six health workers not categorized as skilled in attending labor and delivery. These include Community Health Extension Workers (CHEWs), environmental health technicians, hospital assistants (cleaners), and students. The majority (34%) of the staff in these PHCs have no medical training[50]. Fewer than half of pregnant women in the State access pregnancy care at least four times as recommended, and only about a third access facility based intra-partum care and/or skilled attendance at birth[50]. Cesarean sections are not conducted in PHCs in the study setting.

PHC Selection

Quantitative arm: We selected 10 of the 114 priority PHCs using purposive sampling technique. The 10 had the most deliveries per day on the average in the six-month period prior to starting the study, thus suited to provide rich information on our study objective. They also had labor wards and lying-in wards. The budget for the study dictated the number of health facilities chosen.

Qualitative arm: We selected three of the 10 priority PHC in the quantitative arm of the study. The three had the most deliveries per day on the average. They also had labor wards and lying-in wards. The intensity of the childbirths in the three PHCs and over the study period, will illuminate the barriers and facilitators of early initiation of breastfeeding in public PHCs in the region[51].

Subject Selection

We included all mothers who delivered in the selected PHCs and gave their consent to take part in the study in both arms of the study. For the qualitative arm: we also recruited all consenting healthcare providers who attended deliveries; and the mothers with a live birth for interviews.

Instrument and data collection

For peer review only

Quantitative arm: We recruited and trained nurses and nurse-midwives who are not a part of the health facility staff to complete the assessment tool. They observed and documented the time of events from when a pregnant woman in labor entered the health facility to when she leaves after childbirth or referral. During data collection, they only observed and did not take part or comment in the care offered to the pregnant woman. We only told them to intervene or offer help during a life-threatening situation to the mother and/or baby. The data collectors used separate assessment tools in cases of twin deliveries.

The health facility staff and pregnant women knew of the nurse and nurse-midwives but were unaware of what aspect of care was being observed. Data on the cadre of the health worker, events during the first to third stage of labor, and newborn care activities in the first hour after birth were collected. The nurses and nurse-midwives were available for 24hours in each of the health facilities throughout the study period on a shift schedule. They observed all deliveries.

Qualitative arm: We also recruited and trained female nurses and midwives who are not part of the health facility staff to observe and complete the assessment tool under this arm of the study. Observation for each pregnant woman started during the second stage of labor and ended one hour after childbirth under this arm of the study. The data collector then interviewed the mother and attendant health worker, after the observation period. The assessment tool used for the observation is an extract from the tool used for the quantitative arm. For the mothers and birth attendants we asked questions around how they felt about the childbirth process and knowledge of when newborn babies should breastfeed during the interview. We also asked the mothers why they chose to or not to breastfeed their newborns within the hour depending on if they did or not. Also, we asked the attending healthcare workers why they think breastfeeding occurred or did not occur within the first hour for each newborn. We also asked the attending healthcare workers about the strategies they used to encourage mothers to breastfeed their newborns within the hour. The data collectors attended every birth in the health facilities and interviewed respondents who could not speak or understand English in Hausa. They interviewed the mother and attending healthcare worker separately. The mothers' interviews occurred at their bedside in the lying-in ward. The data collectors excused relatives or visitors in the lying-in room during interviews.

Analysis

For peer review only

Quantitative arm: We analyzed the data using SPSS version 23. We present nominal variables as percentages. We also determined associations and relative risks between initiation of breastfeeding and predictor categorical variables using two-by-two contingency tables. To assess significant associations, we used Fisher's or Pearson's Chi-Squared test as appropriate

Qualitative arm: We transcribed the interviews from their audio files and analyzed the data with Saturate, an online qualitative software. Two people on the team analyzed a subset of the data and generated codes. The two people then came together to review and agree on the codes generated and their meaning, eliminating less useful codes. The two reviewers held regular face-to-face meetings to discuss their codes. We then analyzed the rest of the dataset using the agreed code-framework developed by the two. We generated themes from the codes using an inductive approach.

Patient and Public Involvement Statement

Resources for patient and public involvement statement was unavailable, so we could not involve patients. The development and dissemination of a policy brief of the study findings will involve patients.

RESULT

Respondents' Profile

All pregnant women recruited under the two arms of the study consented to be observed. Under the quantitative arm, we observed 393 pregnant women. Most (54%) were between 15 and 24 years old with a median age of 23 years. A quarter were below the age of 20, half below the age of 23, and three quarters below the age of 30. The childbirths were through spontaneous vaginal delivery. Twin delivery occurred in only six (1.5%) cases. Also, 39% (95% CI: 34%-44%) of the new mothers did not breastfeed their newborns in the first hour after delivery. Thirty-three health workers attended the deliveries under this arm of the study. The number of deliveries attended by each health worker ranged from one to thirty-seven, with an average of 12 (S.D: 10) deliveries each and a median of eight during the observation period. At least two health workers attended about 61% of the 393 deliveries. Also, Junior Community Health Extension Workers (JCHEWs) attended the majority (36%) of the 393 deliveries (Table 1).

Table 1. Pregnant Women's Profile

For peer review only - http://bmjopen.bmj.com/site/about/guidelines.xhtml

	Quantitative arm<br>%<br>n=393	Qualitative arms<br>%<br>n=27
Age		
<15	0	3
15 - 24	53	63
25 - 34	36	30
35+	11	4
Gestational age in weeks		
Mean	41 (SD=12)	38(SD=0.6)
Mode	38	38
Median	38	38
Parity		
Nulliparous (first pregnancy above 28wks gestational age (GA))	20	33
Multiparous (more than first pregnancy above 28wk GA)	80	67
Initiated breastfeeding within the first hour after birth		
no	39	37
yes	61	63
Health worker who attended pregnant woman's labor and delivery		
nurse/midwife	4	0
junior community health extension worker	36	28
community health extension worker	18	15
environmental health assistant/technician/officer	28	19
hospital assistant	10	19
nutritionists/dieticians/students	4	19
Sex of health worker that attended pregnant woman's labor and delivery		
male	1	0
female	99	100

Under the qualitative arm, 27 pregnant women were delivered of their babies by 16 health workers. The pregnant women consented to be observed and interviewed. The 16 health workers also consented to be interviewed after the observation. Most (63%) of the pregnant women were between 15 and 24 years old, with a median age of 22 years. A quarter were below 20 years of age and a quarter above 30 years of age. None had a twin delivery. Also, 37% (95% CI: 19%-56%) of the mothers did not breastfeed the newborn within one hour of birth under this arm of the

For peer review only

study. We also interviewed all 27 mothers and 16 health workers one hour after childbirth. Of the 16 health care workers interviewed under the qualitative arm, most (63%) were hospital assistants. About a third (33%) were Community Health Extension Workers (CHEWS), 17% (2) were students, 6% (1) were nutritionist, and 6% (1) environmental health technician. Of the 27 deliveries under this arm of the study, in 44% of cases, the attending health care worker was assisted by another health worker (Table 1).

Knowledge of time to initiate breastfeeding

The qualitative arm shows that health workers know when mothers should breastfeed newborns. Ninety-two percent of them responded that breastfeeding should start between zero to sixty minutes (Table 2).

Table 2. Health Workers' Response to When Breastfeeding Should Start.

**Attending health care workers interviewed under qualitative arm**	**Frequency**	**Percent** *(n=16)*
Response to when breastfeeding should start		
Immediately after delivery	9	54
**10 - 30 minutes after delivery**	2	13
**30 - 60 minutes after delivery**	1	8
**0 - 60 minutes after delivery**	3	17
**0 - 24 hours after delivery**	1	8

They attributed their knowledge to on-the-job training sessions on newborn care with visiting clinical-mentors. They believe their knowledge is sustained via peer-to-peer discussions while on the job and during staff meetings. One Junior Community Health Extension Worker (JCHEW) said:

"breast feeding should commence immediately after birth …we have clinical mentors that visit and remind us of these things. We also have staff meetings where we remind ourselves of these practices" (birth attendant 1)

The word "immediately" is more used by lower cadre health workers to describe when breastfeeding should start. The CHEWs are more specific about when breastfeeding should start. They also link the time breastfeeding should start with its benefits. The quote below is a typical response from a Senior CHEW when she was asked when breastfeeding should start.

For peer review only - http://bmjopen.bmj.com/site/about/guidelines.xhtml

"It is very important because it helps the child to suck the yellowish nutrient in the breast milk. It boosts the child's immunity. It also helps the mother's uterus to shrink and close …helping to stop bleeding. It should start by 30min to 1hour after delivery" (birth attendant 2).

Barriers to early breastfeeding

Birth attendants' unwillingness or inability to accommodate mothers' safe traditional practices

The quantitative study shows that pregnant women denied safe traditional birth practices such as praying or reading religious texts during the second and third stages of labor are five times more likely not to breastfeed within the first hour (RR=4.5, 95% CI=1.2-17.1) compared to pregnant women allowed these practices (Table 3).

Table 3: Association Between Early Breastfeeding and Predictor Variables in the Study

Variable	Response	Women who didn't initiate breastfeeding within 1hr after birth		
		% n=154	*Relative risk 95% CI*	*p value*
Birth attendant had received training on newborn care	No	49	1.2 (0.9-1.5)	0.2
	Yes	51		
There were delays in providing care	No	90	0.7 (0.5-1.03)	0.2
	Yes	10		
Communication was easy and frequent between woman and birth attendant	No	2	0.7 (0.3-1.8)	0.5
	Yes	98		
Poor staff attitude	No	96	0.7 (0.4-1.1)	0.2
	Yes	4		
Woman denied some safe traditional childbirth practices*	No	99	4.5 (1.2-17.1)	0.003***
	Yes	1		
Birth attendant determined the birth position	No	41	1 (0.8-1.3)	>0.9
	Yes	59		
Woman was allowed to give birth in the position she preferred**	No	37	0.9 (0.7-1.2)	0.5
	Yes	63		
Woman encouraged to consume fluids/food at least once during labor	No	16	2.1 1.5-3.0	0.001
	Yes	84		
Mother and newborn kept in the same room after delivery (rooming-in)	No	2	2.6 2.3-2.9	0.059***
	Yes	98		
Mother had skin-to-skin contact with newborn in the	No	45	2.3	<0.001

For peer review only

first hour after birth	Yes	55	1.8-2.8	
Woman had blood loss greater than 500mls during labor and delivery	No	99	1.17<br>0.2-5.9	>0.9***
	Yes	1		
The labor room was clean and comfortable	No	18	0.98<br>(0.7-1.4)	0.9
	Yes	82		

**n=154, **n=150, ***Fishers* X^2

When mother's state of health after childbirth isn't an issue, a typical response given by some mothers for not breastfeeding in the first hour was the need to first wash the breast or have a bath to feel clean.

"he (the baby) has to exercise patience until we get home (before he is breastfed). I can't breastfeed him before I take my bath" (mother 1)

The quantitative study suggests that the mother's need to be clean may not be influenced by the cleanliness of the environment. Initiating breastfeeding is not related to the cleanliness and comfort provided by the delivery room. Mothers who had their babies when the labor room was clean and comfortable were just as likely not to initiate breastfeeding early as those who had their babies when the labor room was not (RR=0.98; 95% CI 0.7-1.4; p=0.9).

Poor management of mothers' post-delivery state of health

Post-delivery pains and fatigue are barriers to breastfeeding within the first hour after birth. Even when mothers show a good knowledge of when breastfeeding should start, some still express the need to regain strength and wellness first before they breastfeed the newborn.

"breastfeeding should commence immediately after birth… I did not commence it because I was feeling after pains. The health worker said she will bring the baby to suck. I told her to allow me to have some relief" ZA (mother 2)

The quantitative study shows that pregnant women not encouraged to consume fluids or food at least once during labor are twice as likely not to breastfeed within the first hour compared with those encouraged to do so (RR=2.1; 95% CI=1.5-3; p=0.001). There is no evidence from the study that blood loss greater than 500mls during labor and delivery influence early breastfeeding (RR=1.17; 95% CI=0.2-5.9; p=1).

Human resource shortages

For peer review only

The qualitative study shows that shortage of health workers in PHCs introduces a delay in carrying out newborn care activities. Sometimes, health workers have to attend to other ill patients when there is no one else to assist. When we asked some health workers why a mother under their care did not breastfeed early, a typical response given was:

““You see, if you have someone that will assist you, you will assign the person to carry the baby to the mother and initiate the breastfeeding, or weigh the child, or apply chlorhexidine to the baby’s cord or any other thing needed while you continue with the remaining work and management of others …but most of the time you are on duty alone. It is because we have shortage of manpower here in this facility” (birth attendant 3)

The human resource shortage also affects rooming-in in the PHCs. Placing the mother and newborn in the same room after delivery is rooming-in[52]. Sometimes, the health worker has to clean and make this room ready for the mother and newborn. When there is a shortage of staff, this delays the transfer of the mother and newborn to the rooming-in room, and affects early breastfeeding.

“I wanted to transfer the mother and baby to the postnatal ward… before she commences breastfeeding (of the newborn). The (lying-in) room is not set (for use yet). I have to clean and make the room so she is comfortable to commence breastfeeding” (birth attendant 4)

Ineffective rooming-in practices

When rooming-in happens, the quantitative study reveals that mothers who do not have skin-to skin contact with their newborns in the first hour after birth are twice as likely not to breastfeed early, compared to mothers who did (RR=2.3, 95% CI=1.8-2.8; p<0.001). Just keeping the mother and newborn in the same room (rooming-in) without skin-to-skin contact has no influence on early breastfeeding (RR=2.6; 95% CI=2.3-2.9; p=0.059).

Lack of privacy and proper visiting-hour policy in the PHCs

The PHCs have open rooming-in rooms that doesn’t guarantee privacy. There are also no defined visiting hours in the PHCs. The qualitative arm of the study shows that male and female relatives visit the new mother in the hospital after childbirth. During the visit, the relatives pray for the newborn baby and congratulate the mother. Some relatives sit around after prayers for long. When relatives come visiting, the mothers have to dress up to receive them and do not breastfeed

For peer review only

during this time. The birth attendants also delay supporting the mother to breastfeed. One birth attendant suggested that addressing the issue puts them in bad light in the community.

"you know, the people in the community have a unique character or attitude. The moment you try to talk to them about this kind of issue they feel you are molesting them or depriving them of coming close to their relatives. They do not know you are trying to ensure their relative (mother and baby) gets what is beneficial to them" (birth attendant 5).

Facilitators of early breastfeeding

Health education during ANC and post-delivery period

From the qualitative study, we find that Antenatal Clinics (ANC) helps pregnant women to learn about breastfeeding newborns within one hour of birth. The knowledge they gain during these clinics influences their behavior after childbirth. Most mothers who practiced early breastfeeding said what they learned from ANC influenced their decision to do so. The typical response they gave is:

"I used to give my children water (after childbirth). I did not know the importance of breastfeeding early. I used to think breast feeding could start at any time of the day (of birth). I started breastfeeding early because the health workers tell us (of the importance of starting breastfeeding immediately after birth) during ANC" (mother 3)

Encouraging and supporting mothers to start breastfeeding after childbirth

From the qualitative study, we find that when the birth attendants encourage some reluctant mothers to breastfeed their newborns within one hour of childbirth; they do.

"I was told (by the health worker) to give (the baby breast to suck early enough). Normally, I won't" (mother 4)

The encouragement process takes the form of a negotiation between the birth attendant and the mother. We find that the information passed by the birth attendants to the mothers around the benefits of early breastfeeding, facilitate early breastfeeding in the PHCs.

"I commenced breastfeeding early because of what the health worker said …she explained how it is important to the health of my baby. That's why I commenced it" (mother 5)

For peer review only

Sometimes, showing the mothers how to place and breastfeed the newborns also facilitates early breastfeeding after childbirth in these health facilities.

"I gently encourage them and tell them to give (breast milk) early… I put the baby on her laps and remove the breast, and demonstrate to her how to breastfeed" (birth attendant 6)

DISCUSSION

In our study setting, we find that close to four out of every 10 newborns do not get breastfed within the first hour of birth. This doubles their risk of dying in the first 28 days of their lives[10,15]. Our estimate is four percentage points lower than what researcher observed in PHCs in Asia[31]. It is also higher than estimates amongst mothers who had spontaneous vaginal deliveries (SVD) in some secondary health facilities; and lower than estimates amongst mothers who had SVD in some tertiary health facilities, even in Nigeria[31,53,54]. The mixed results emphasize the influence of context on the early breastfeeding of newborns in different health facilities. It underscores the need for unique interventions to address the problem.

The northeast region of Nigeria has a shortage of skilled health workers. The Boko-Haram insurgency has made this worse[55–57]. It is also worsened by staff absenteeism in PHCs in the region. Only about 35% of employed staff in PHCs in Gombe are likely to be at work on any given day, for example[22]. Studies suggest that a shortage of human resource and a dominant population of unskilled health care workers affects the quality of newborn care in health facilities[17,58–60]. This is not overall consistent with our study findings. We find that unskilled health workers are dominant in our study setting. Also, we find that human resource shortage is a barrier to early initiation of breastfeeding in the PHCs. We did not find that the skills of the birth attendants influenced the early breastfeeding of newborns. This may be because clinical mentors have trained the birth attendants in our study on newborn care. Educational interventions around support for the breastfeeding of newborns has been found to improve health workers' knowledge, attitude, and compliance with the practice[61,62].

In our study, we find that the mothers denied safe traditional birth practices like praying, reciting religious texts, or reading religious books during deliveries were five times more likely not to breastfeed the newborns than the mothers not denied. This supports the findings from other settings that shows that the unwillingness of birth attendants to accommodate safe traditional birth practices affects mothers' adoption of supportive care[59,63,64]. This may be because mothers

For peer review only

perceive denial of such traditional practices as mistreatment or abuse[59,65]. A study in Norway finds that the recent abuse of women by "both known and unknown" perpetrators affects their breastfeeding behavior[66]. Although in our study, we found no evidence to support this. Instead we find that birth attendants' attitude, rapport skills, and negligence during the delivery period doesn't affect early breastfeeding practice amongst mothers. The discrepancy in findings may be because the women in our study setting perceive or tolerate abuse differently than women in other settings[67].

Washing of the breast with water after childbirth is a hygiene related practiced in parts of the world[68]. In our study, mothers express a strong need to wash the breast and/or have a bath before breastfeeding newborns. This makes the "need for a bath after childbirth" a major theme for why mothers delay breastfeeding newborns in our study setting. Some health workers also agree that mothers should wash their breasts first before breastfeeding newborns after childbirth[18]. Their inability to help the mothers to wash their breasts or have a bath before breastfeeding may be because there is no running water in the health facility. Only 38% of priority PHCs in Gombe have running water[22]. The need to wash the breast may be deeper than the need for hygiene, nonetheless. Washing of the breast or having a bath after childbirth before breastfeeding newborns has traditional and/or religious undertones in other parts of the world[60,69].

The other barriers that influence early breastfeeding of newborn in PHCs that our study finds are post-delivery pains and fatigue within the first hour after birth, delay in skin to skin contact during rooming-in, and poor visiting hour policy in the PHC. These findings reinforces what other studies have reported[5,21,24,33,41,54,60]. The post-delivery fatigue may be because the mother is famished. This may also be due to anemia, infections, thyroid disorders, mood disorders and cardiomyopathy[70] which our study does not assess. The quantitative arm of our study shows that mothers not encouraged to take fluids or eat during the second and third stages of labor are more likely not to breastfeed early after childbirth. There is no evidence from our study that the fatigue may be due to blood loss. We also find that rooming-in is not enough and that skin to skin contact must be deliberate. Mothers that did not have skin to skin contact with their newborns while in the rooming-in room were more likely not to breastfeed than those who did in our study. Our study reemphasizes that the lack of restrictions on relatives visiting the mothers in the post-delivery period impedes breastfeeding[71]. This could be because the mothers don't feel comfortable breastfeeding in front of male relatives or visitors.

For peer review only - http://bmjopen.bmj.com/site/about/guidelines.xhtml

Helping mothers to breastfeed within an hour of birth is an international recommendation practiced by health care providers in most of the world[32,72]. We find that birth attendants in our study setting practice this and the strategy facilitates the early breastfeeding of newborns in public PHCs. This may be because there is an awareness of the recommendation[73]. Our study also reinforces findings from previous studies on the benefits of health education on the timely breastfeeding of newborns[74,75]. In our study, we find that health education received by mothers during ANC and the post-delivery period improves the practice of early initiation of breastfeeding in public PHCs, in our study setting

CONCLUSION

Only about 60% of babies born in public PHCs in Northeast Nigeria get breastfed in the first hour of birth. This means the rest miss important nutrition that saves lives. The stakeholders in the region must increase their focus on improving the breastfeeding practices in public PHCs. This will improve the survival of newborns and impact of their investments. Instituting policies that protect mothers' privacy; and finding innovative ways to accommodate and promote safe traditional practices in the intrapartum and postpartum period in PHCs will improve the early breastfeeding of newborns in these PHCs. Birth attendants in PHCs must also be trained on effective rooming-in to further improve early breastfeeding of newborns in these public PHCs, however.

LIST OF ABBREVIATIONS

PHC (Primary Health Care hospital/facility), ANC (Antenatal Care) VHW (Village Health Workers), CHEWs (Community Health Extension Workers).

DECLARATIONS

Ethics approval and consent to participate

The Gombe State Ministry of Health Ethics Committee granted ethical approval for the qualitative arm of the study (reference no: MOH/ADM/658/VOL.II/104). Mothers and birth attendants also gave their consent to take part in the study before the interviews. The IDEAS team at the London School of Hygiene and Tropical Medicine (LSHTM) got ethical approval for the quantitative arm of the study from LSHTM (reference 6088).

Funding

For peer review only

The study is funded as part of the evaluation of SFH's MNCH2 project being funded by the Bill and Melinda Gates Foundation and implemented in Gombe State. The views and opinions expressed in the paper are those of the authors and do not reflect those of SFH and/or GSPHCDA. The intervention is funded through donor grants received by SFH. The donor did not play a role in the design of this evaluation.

Data Availability

Data for the quantitative arm of the study is in a secured database at the London School of Hygiene and Tropical Medicine. Write to Nasir Umar through nasir.umar@lshtm.ac.uk to place a reasonable request for the anonymized version of it. Also, write to Shobo Olukolade, through shoboolukolade@gmail.com to ask for the anonymized transcripts of the qualitative data.

Competing interest

OGS and PL are consultants working for Society for Family Health (SFH), OI and JA are full-time staff of SFH. GA is the Former Executive Secretary of Gombe State Primary Health Care Development Agency. SFH's program in Northeast Nigeria seeks to improve MNCH outcomes in the general population.

Author Contribution

OGS conceived of the study and developed the original draft of the manuscript. OGS analyzed the quantitative data. OGS and PL analyzed the qualitative data. NU, GA, JA, and OI reviewed, edited, and made significant contributions to the development of the final manuscript. All authors read and approved the final manuscript.

Acknowledgement

For peer review only

We thank all the staff of SFH MNCH2 project in Gombe, particularly Mrs. Magdalene Okolo for their contributions to the study. We also thank the field staff who conducted the observations and interviews. We also thank our partners, the members of the IDEAS team from the London School of Hygiene and Tropical Medicine, for leadership in designing and implementing the quantitative arm of the study.

REFERENCES

1. World Health Organization. *Reaching the every newborn national 2020 milestones: country progress, plans and moving forward*. http://apps.who.int/iris/bitstream/handle/10665/255719/9789241512619-eng.pdf?sequence=1 (2017).
2. Edmond, K. M. *et al.* Delayed Breastfeeding Initiation Increases Risk of Neonatal Mortality. *Pediatrics* **117**, e380–e386 (2006).
3. Mullany, L. C. *et al.* Breast-Feeding Patterns, Time to Initiation, and Mortality Risk among Newborns in Southern Nepal. *J Nutr* **138**, 599–603 (2008).
4. World Health Organization. *Every newborn: an action plan to end preventable deaths*. (World Health Organization, 2014).
5. WHO | Early initiation of breastfeeding to promote exclusive breastfeeding. *WHO* http://www.who.int/elena/titles/early_breastfeeding/en/.
6. WHO | Newborn care at birth. *WHO* http://www.who.int/maternal_child_adolescent/newborns/care_at_birth/en/.
7. Debes, A. K., Kohli, A., Walker, N., Edmond, K. & Mullany, L. C. Time to initiation of breastfeeding and neonatal mortality and morbidity: a systematic review. *BMC Public Health* **13**, S19 (2013).

For peer review only

8. UNICEF & WHO. *Capture the moment: early initiation of breastfeeding : the best start for every newborn*. (2018).

9. Friedrich, M. J. Early Initiation of Breastfeeding. *JAMA* **320**, 1097–1097 (2018).

10. al, K. J., et. Timing of breastfeeding initiation and exclusivity of breastfeeding during the first month of life: effects on neonatal mortality and morbidity--a ... - PubMed - NCBI. https://www.ncbi.nlm.nih.gov/pubmed/24894730.

11. Federal Ministry of Health. Nigeria Every Newborn Action Plan: A plan to end preventable newborn deaths in Nigeria. (2016).

12. Anyamele, O., Akanegbu, B., Assad, J.-C. & Ukawuilulu, J. Differentials in Infant and Child Mortality in Nigeria: Evidence from Pooled 2003 and 2008 DHS Data. *Advances in Management and Applied Economics* **7**, 73–96 (2017).

13. Morakinyo, O. M. & Fagbamigbe, A. F. Neonatal, infant and under-five mortalities in Nigeria: An examination of trends and drivers (2003-2013). *PLOS ONE* **12**, e0182990 (2017).

14. Tura, G., Fantahun, M. & Worku, A. The effect of health facility delivery on neonatal mortality: systematic review and meta-analysis. *BMC Pregnancy Childbirth* **13**, 18 (2013).

15. Khan, J., Vesel, L., Bahl, R. & Martines, J. C. Timing of Breastfeeding Initiation and Exclusivity of Breastfeeding During the First Month of Life: Effects on Neonatal Mortality and Morbidity—A Systematic Review and Meta-analysis. *Matern Child Health J* **19**, 468–479 (2015).

16. Takahashi, K. *et al.* Prevalence of early initiation of breastfeeding and determinants of delayed initiation of breastfeeding: secondary analysis of the WHO Global Survey. *Scientific Reports* **7**, 44868 (2017).

For peer review only

17. Haile, Z. T. *et al.* The Moderating Effect of Urban Versus Rural Residence on the Relationship Between Type of Birth Attendant and Early Initiation of Breastfeeding in Ghana. *J Hum Lact* **34**, 810–820 (2018).

18. Bee, M., Shiroor, A. & Hill, Z. Neonatal care practices in sub-Saharan Africa: a systematic review of quantitative and qualitative data. *Journal of Health, Population and Nutrition* **37**, 9 (2018).

19. Sobel, H. L., Silvestre, M. A. A., Iii, J. B. V. M., Oliveros, Y. E. & Nyunt-U, S. Immediate newborn care practices delay thermoregulation and breastfeeding initiation. *Acta Paediatrica* **100**, 1127–1133 (2011).

20. Adewuyi, E. O., Zhao, Y., Khanal, V., Auta, A. & Bulndi, L. B. Rural-urban differences on the rates and factors associated with early initiation of breastfeeding in Nigeria: further analysis of the Nigeria demographic and health survey, 2013. *International Breastfeeding Journal* **12**, 51 (2017).

21. Berde, A. S. & Yalcin, S. S. Determinants of early initiation of breastfeeding in Nigeria: a population-based study using the 2013 demograhic and health survey data. *BMC Pregnancy and Childbirth* **16**, 32 (2016).

22. Marchant, T. *Change in maternal and newborn health care interactions between families and frontline workers. Report from Gombe State, Nigeria, 2012-2015*. https://ideas.lshtm.ac.uk/wp-content/uploads/2017/08/Marchant_IDEAS_Gombe_State_Follow-upSurvey_2016.pdf (2016).

23. Thulier, D. & Mercer, J. Variables Associated With Breastfeeding Duration. *Journal of Obstetric, Gynecologic & Neonatal Nursing* **38**, 259–268 (2009).

For peer review only

24. Ahmed, A. E. & Salih, O. A. Determinants of the early initiation of breastfeeding in the Kingdom of Saudi Arabia. *International Breastfeeding Journal* **14**, 13 (2019).

25. Liben, M. L. & Yesuf, E. M. Determinants of early initiation of breastfeeding in Amibara district, Northeastern Ethiopia: a community based cross-sectional study. *International Breastfeeding Journal* **11**, 7 (2016).

26. Delivery care. *UNICEF DATA* https://data.unicef.org/topic/maternal-health/delivery-care/.

27. Bohren, M. A. *et al.* Facilitators and barriers to facility-based delivery in low- and middle-income countries: a qualitative evidence synthesis. *Reproductive Health* **11**, 71 (2014).

28. Campbell, O. M. & Graham, W. J. Strategies for reducing maternal mortality: getting on with what works. *The Lancet* **368**, 1284–1299 (2006).

29. WHO | Early initiation of breastfeeding. *WHO* http://www.who.int/elena/titles/commentary/early_breastfeeding/en/.

30. Forster, D. A. & McLachlan, H. L. Breastfeeding Initiation and Birth Setting Practices: A Review of the Literature. *Journal of Midwifery & Women's Health* **52**, 273–280 (2007).

31. Karim, F. *et al.* Initiation of breastfeeding within one hour of birth and its determinants among normal vaginal deliveries at primary and secondary health facilities in Bangladesh: A case-observation study. *PLOS ONE* **13**, e0202508 (2018).

32. WHO | Protecting, promoting and supporting breastfeeding in facilities providing maternity and newborn services. *WHO* http://www.who.int/elena/titles/full_recommendations/breastfeeding-support/en/.

33. Sharma, I. K. & Byrne, A. Early initiation of breastfeeding: a systematic literature review of factors and barriers in South Asia. *International Breastfeeding Journal* **11**, 17 (2016).

For peer review only

34. DiGirolamo, A. M., Grummer-Strawn, L. M. & Fein, S. Maternity Care Practices: Implications for Breastfeeding. *Birth* **28**, 94–100 (2001).

35. Fedreal Ministry of Health. Nigeria Investment case for reproductive, maternal, newborn, child, adolescent health and nutrition: 2017 - 2030. (2017).

36. The Maternal and Neonatal Health Project in North East Nigeria 2012 – 2016 | Society for Family Health, Nigeria. http://www.sfhnigeria.org/the-maternal-and-neonatal-health-project-in-north-east-nigeria-2012-2016/.

37. State Accountability and Quality Improvement Project. *Pact* https://www.pactworld.org/country/nigeria/project.

38. Strengthening the healthcare system in north-east Nigeria - a priority for WHO | WHO | Regional Office for Africa. https://www.afro.who.int/news/strengthening-healthcare-system-north-east-nigeria-priority-who.

39. Makinde, O. A., Sule, A., Ayankogbe, O. & Boone, D. Distribution of health facilities in Nigeria: Implications and options for Universal Health Coverage. *The International Journal of Health Planning and Management* https://onlinelibrary.wiley.com/doi/abs/10.1002/hpm.2603 (2018) doi:10.1002/hpm.2603.

40. Nigeria Health Facility Registry. https://hfr.health.gov.ng/facilities/search?_token=r2vSeMamYxcYqkwMtdRF67CgGTWkRNkpYS6QoUQV&facility_type_id=1&state_id=115&lga_id=&ward_id=0&facility_level_id=2&ownership_id=1&operational_status_id=0&registration_status_id=0&license_status_id=0&geo_codes=0&service_type=0&service_category_id=0&facility_name=.

41. Bbaale, E. Determinants of Early Initiation, Exclusiveness, and Duration of Breastfeeding in Uganda. *J Health Popul Nutr* **32**, 249–260 (2014).

42. Li, R., Scanlon, K. S. & Serdula, M. K. The Validity and Reliability of Maternal Recall of Breastfeeding Practice. *Nutr Rev* **63**, 103–110 (2005).

43. Kawulich, B. B. Participant Observation as a Data Collection Method. *Forum Qualitative Sozialforschung / Forum: Qualitative Social Research* **6**, (2005).

44. Altmann, J. Observational Study of Behavior: Sampling Methods. *Behaviour* **49**, 227–267 (1974).

45. Schoonenboom, J. & Johnson, R. B. How to Construct a Mixed Methods Research Design. *Kolner Z Soz Sozpsychol* **69**, 107–131 (2017).

46. Denzin, N. K. & Lincoln, Y. S. *The SAGE Handbook of Qualitative Research*. (SAGE, 2011).

47. Gombe State Government. Gombe State Nigeria: History. *Gombe State* http://gombestate.gov.ng/history-2/.

48. Federal Ministry of Health. Federal Republic of Nigeria Draft National Human Resources for Health Strategic Plan 2008 - 2012. (2012).

49. Alenoghena, I. O., Isah, E. C. & Isara, A. R. Availability and Type of Human Resource for Health in Public Primary Health Care Facilities in Selected Communities, Edo State. *Journal of Community Medicine and Primary Health Care* **28**, 11–18 (2016).

50. Marchant, D. T. Maternal and newborn health care. Baseline findings from Gombe State, Nigeria. https://ideas.lshtm.ac.uk/report/mnh-care-baseline-gombe-state/ (2013) doi:10.17037/PUBS.01126638.

51. Patton, M. Designing Qualitative Studies. in *Qualitative evaluation and research methods* 169–186 (SAGE, 1990).

52. Jaafar, S. H., Ho, J. J. & Lee, K. S. Rooming-in for new mother and infant versus separate care for increasing the duration of breastfeeding. *Cochrane Database Syst Rev* CD006641–CD006641 (2016) doi:10.1002/14651858.CD006641.pub3.

For peer review only

53. Örün, E. *et al.* Factors associated with breastfeeding initiation time in a Baby-Friendly Hospital. *The Turkish Journal of Pediatrics* **52**, 10–16 (2010).

54. Awi, D. D. & Alikor, E. a. D. Barriers to timely initiation of breastfeeding among mothers of healthy full-term babies who deliver at the University of Port Harcourt Teaching Hospital. *Nigerian Journal of Clinical Practice* **9**, 57-64–64 (2006).

55. Federal Ministry of Health. National Human Resource for Health Strategic Plan: 2008 - 2012. (2008).

56. Premium Times Nigeria. Gombe govt. waives retirement for 100 nurses, midwives due to manpower shortage. https://www.premiumtimesng.com/news/more-news/230165-gombe-govt-waives-retirement-100-nurses-midwives-due-manpower-shortage.html (2017).

57. Omoluabi, E. *Needs Assessment of the Nigerian Health Sector*. https://nigeria.iom.int/sites/default/files/newsletter/ANNEX%20XXIV%20Needs%20Assessment%20of%20the%20Nigeria%20health%20Sector.pdf (2014).

58. Aluko, J. O., Anthea, R. & Marie Modeste, R. R. Manpower capacity and reasons for staff shortage in primary health care maternity centres in Nigeria: a mixed-methods study. *BMC Health Services Research* **19**, 10 (2019).

59. Bohren, M. A. *et al.* The Mistreatment of Women during Childbirth in Health Facilities Globally: A Mixed-Methods Systematic Review. *PLoS Med* **12**, e1001847 (2015).

60. Majra, J. P. & Silan, V. K. Barriers to Early Initiation and Continuation of Breastfeeding in a Tertiary care Institute of Haryana: A Qualitative Study in Nursing Care Providers. *J Clin Diagn Res* **10**, LC16–LC20 (2016).

61. Balogun, O. O. *et al.* Health Facility Staff Training for Improving Breastfeeding Outcome: A Systematic Review for Step 2 of the Baby-Friendly Hospital Initiative. *Breastfeeding Medicine* **12**, 537–546 (2017).

For peer review only

62. Okolo, S. N. & Ogbonna, C. Knowledge, attitude and practice of health workers in Keffi local government hospitals regarding Baby-Friendly Hospital Initiative (BFHI) practices. *Eur J Clin Nutr* **56**, 438–441 (2002).

63. Mannava, P., Durrant, K., Fisher, J., Chersich, M. & Luchters, S. Attitudes and behaviours of maternal health care providers in interactions with clients: a systematic review. *Globalization and Health* **11**, 36 (2015).

64. Chatters, L. M. Religion and Health: Public Health Research and Practice. *Annual Review of Public Health* **21**, 335–367 (2000).

65. Beck, C. T. A Secondary Analysis of Mistreatment of Women During Childbirth in Health Care Facilities. *Journal of Obstetric, Gynecologic & Neonatal Nursing* **47**, 94–104 (2018).

66. Sørbø, M. F., Lukasse, M., Brantsæter, A.-L. & Grimstad, H. Past and recent abuse is associated with early cessation of breast feeding: results from a large prospective cohort in Norway. *BMJ Open* **5**, e009240 (2015).

67. Maya, E. T. *et al.* Women's perspectives of mistreatment during childbirth at health facilities in Ghana: findings from a qualitative study. *Reproductive Health Matters* **26**, 70–87 (2018).

68. Khan, M. E. Shaping Demand and Practices to Improve Family Health Outcomes: Findings from a Quantitative Survey, Volume 3: Bihar. 221.

69. Tawiah-Agyemang, C., Kirkwood, B. R., Edmond, K., Bazzano, A. & Hill, Z. Early initiation of breast-feeding in Ghana: barriers and facilitators. *Journal of Perinatology* **28**, S46–S52 (2008).

70. Atkinson, L. S. & Baxley, E. G. Postpartum fatigue. *Am Fam Physician* **50**, 113–118 (1994).

For peer review only

71. Saxena, M., Srivastava, A., Dwivedi, P. & Bhattacharyya, S. Is quality of care during childbirth consistent from admission to discharge? A qualitative study of delivery care in Uttar Pradesh, India. *PLOS ONE* **13**, e0204607 (2018).

72. UNICEF & World Health Organization. Country Experiences with the Baby-Friendly Hospital Initiatiive. Compendium of Case Studies from Around the World. (2017).

73. Kakrani, V. A., Rathod (Waghela), H. K., Mammulwar, M. S. & Bhawalkar, J. S. Awareness about "Ten Steps for Successful Breastfeeding" among Medical and Nursing Students. *Int J Prev Med* **6**, (2015).

74. Guise, J.-M. *et al.* The Effectiveness of Primary Care-Based Interventions to Promote Breastfeeding: Systematic Evidence Review and Meta-Analysis for the US Preventive Services Task Force. *Ann Fam Med* **1**, 70–78 (2003).

75. de Oliveira, M. I. C., Camacho, L. A. B. & Tedstone, A. E. Extending Breastfeeding Duration Through Primary Care: A Systematic Review of Prenatal and Postnatal Interventions. *J Hum Lact* **17**, 326–343 (2001).

For peer review only
